# Gene therapy breakthroughs in ALS: a beacon of hope for 20% of ALS patients

**DOI:** 10.1186/s40035-025-00477-6

**Published:** 2025-04-16

**Authors:** Qingjian Xie, Kezheng Li, Yinuo Chen, Yaojia Li, Wenhua Jiang, Wen Cao, Huan Yu, Dongsheng Fan, Binbin Deng

**Affiliations:** 1 Department of Rehabilitation, The First Affiliated Hospital of Wenzhou Medical University, Wenzhou, China; 2 Department of Neurology, First Affiliated Hospital of Wenzhou Medical University, Wenzhou, 32500 China; 3 First School of Clinical Medicine, Wenzhou Medical University, Wenzhou, China; 4 Zhejiang Key Laboratory of Intelligent Cancer Biomarker Discovery and Translation, First Affiliated Hospital of Wenzhou Medical University, Wenzhou, China; 5 Department of Pediatrics, Second Affiliated Hospital and Yuying Children’S Hospital of Wenzhou Medical University, Wenzhou, China; 6 Department of Neurology, Peking University Third Hospital, Beijing, China

**Keywords:** Amyotrophic lateral sclerosis, Gene therapy, Gene therapy vectors, Gene targeting sites

## Abstract

Amyotrophic lateral sclerosis (ALS) is a fatal motor neuron disease that remains incurable. Although the etiologies of ALS are diverse and the precise pathogenic mechanisms are not fully understood, approximately 20% of ALS cases are caused by genetic factors. Therefore, advancing targeted gene therapies holds significant promise, at least for the 20% of ALS patients with genetic etiologies. In this review, we summarize the main strategies and techniques of current ALS gene therapies based on ALS risk genes, and review recent findings from animal studies and clinical trials. Additionally, we highlight ALS-related genes with well-understood pathogenic mechanisms and the potential of numerous emerging gene-targeted therapeutic approaches for ALS.

## Introduction

Amyotrophic lateral sclerosis (ALS) is a progressive and heterogeneous neurodegenerative disease affecting both upper motor neurons and lower motor neurons, ultimately leading to muscle weakness, paralysis, and death [1]. Traditional treatments for ALS are primarily focused on disease modification and improving clinical care. For instance, the anti-glutamate drug riluzole has been shown in clinical trials to improve survival in ALS patients, and non-invasive ventilation can enhance survival and quality of life [2–4]. However, these therapies cannot cure ALS completely, highlighting the urgent need for the development of more effective treatments.

Over the past two decades, significant advances have been made in the genetics and molecular pathology of ALS. Advanced genetic techniques such as sequencing have accelerated the identification of ALS-related pathogenic genes, and a substantial number of genetic loci and mechanisms associated with ALS pathogenesis have been revealed [5]. Among these, genes such as Chromosome 9 Open Reading Frame 72 (*C9orf72*), Superoxide dismutase 1 (*SOD1*), TAR DNA-binding protein (*TARDBP*), and Fused in sarcoma (*FUS*) are the most widely studied. Additionally, recent studies have identified risk genes associated with ALS, including Valosin containing protein (*VCP*), TANK-binding Kinase 1 (*TBK1*), and NIMA Related Kinase 1 (*NEK1*) [6]. Proteins encoded by these genes often form aggregates, which impact intracellular RNA and protein quality control, and participate in the pathogenesis of ALS through various mechanisms [7]. Based on this background, the focus of ALS treatment research has gradually shifted towards gene therapy.

Gene therapy aims to fundamentally alter the course of a disease by precisely modifying, replacing, or suppressing pathogenic genes, enabling sustained expression of therapeutic genes or "transgenes". This approach offers a novel treatment option for diseases that are currently untreatable by conventional methods [8]. For ALS, at least five antisense oligonucleotides (ASOs) are currently undergoing clinical trials for ALS [9]. Ongoing research indicates that the RNA interference (RNAi)-based ALS therapies also hold promise for future clinical applications [9]. Although Clustered-Regularly-Interspaced-Short-Palindromic-Repeats-CRISPR-Associated-Protein-9 (CRISPR-Cas9) gene editing is primarily applied in clinical trials for hematological diseases and cancers, it also shows considerable potential in ALS gene therapy [10]. Furthermore, adeno-associated virus (AAV) vectors have been explored for its ability to reduce SOD1 levels in phase I trials [11].

However, several challenges need to be addressed in the development of new therapies, such as determining the optimal time to start treatment, developing and optimizing effective drug delivery systems, controlling immune responses, and translating animal models to clinical applications [9, 12]. In this review, we provide an overview of general gene therapy strategies, elucidate the specific applications of gene vectors in ALS, categorize and summarize risk-associated genes involved in various ALS mechanisms, and analyze the current status and challenges of ALS to offer insights and guidance for future research and clinical applications of ALS gene therapy.

## ALS gene therapy strategies

**Fig. 1:**
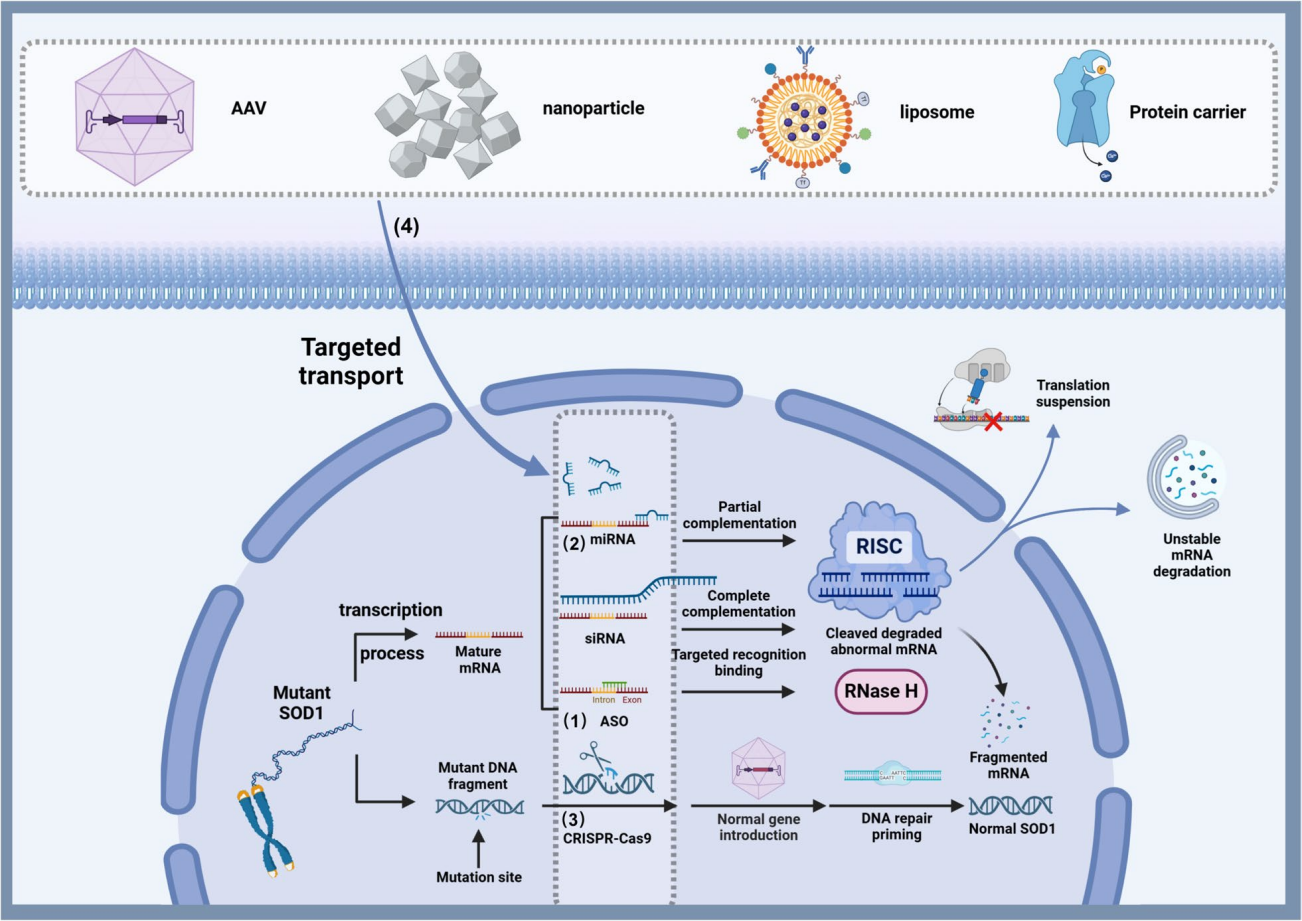
Illustration of ALS gene therapy strategies using *SOD1* mutations as an example. (1) ASOs are short, synthetic RNA or DNA strands, typically designed to be complementary to specific mRNA sequences within cells. Upon binding to their target mRNA, ASOs recruit RNase H, which recognizes the RNA–DNA or RNA-RNA duplex formed by the ASO and its target mRNA. RNase H then cleaves the mRNA strand of the duplex, reducing the production of the protein encoded by the mRNA. (2) siRNAs are completely complementary to their target mRNAs, leading RISC to directly cleave the target mRNAs, resulting in their degradation. miRNAs, on the other hand, are partially complementary to their target mRNAs, typically binding to the 3′ untranslated region (3′ UTR), where they primarily inhibit translation or cause mRNA destabilization and degradation. (3) CRISPR gene editing employs a gRNA to direct the Cas9 enzyme to specific locations in the DNA. After binding to the target DNA sequence, Cas9 induces double-strand breaks in the DNA. The cell then initiates repair processes, which can be harnessed to introduce changes to the gene sequence, such as knocking out harmful genes or correcting mutations. (4) Various gene delivery vehicles, including AAV, nanoparticles, liposomes, and protein carriers, are used to enhance the precise delivery of ASOs, RNAi agents, CRISPR-Cas9 agents, neurotrophic factors, and other therapeutic agents, thereby improving their transport efficiency. Figure Created with BioRender.com

Gene therapy for ALS involves delivering genetic material to cells with the aim of introducing functional copies of dysfunctional genes, trophic factors, or other disease-modifying genes. It may also involve silencing the expression of harmful genes. This is particularly crucial in ALS, where genetic mutations can lead to neurodegeneration [13] (Fig. 1). The application of various gene therapy strategies and related clinical studies are presented in Table 1.

**Table 1:** Ongoing or completed clinical trials of gene therapies for ALS

Gene therapy strategy	Study title	Method	Outcome variable	Result
ASO	Phase 1–2 Trial of Antisense Oligonucleotide Tofersen for *SOD1* ALS [62] ClinicalTrials.gov number, NCT02623699	In each dose group (20, 40, 60, or 100 mg), participants were randomly assigned in a 3:1 ratio to receive either 5 doses of toferson or placebo via intrathecal administration over 12 weeks	Primary outcomes: Safety and pharmacokinetics Secondary outcomes: Changes in cerebrospinal fluid (CSF) SOD1 concentration relative to baseline on day 85	Reduced CSF SOD1 levels at peak levels within 12 weeks
	Trial of Antisense Oligonucleotide Tofersen for *SOD1* ALS [63] ClinicalTrials.gov number, NCT02623699	In this Phase 3 trial, adults with *SOD1* ALS were randomized in a 2:1 ratio to receive either 8 doses of tofersen (100 mg) or placebo over 24 weeks	Primary outcome: Change from baseline to Week 28 in ALS Functional Rating Scale-Revised (ALSFRS-R) total score among participants predicted to have faster disease progression Secondary outcome: Changes in total SOD1 protein concentration in CSF, concentration of neurofilament light chain (NfL) in plasma, forced vital capacity, and handheld dynamometry (HHD) of 16 muscle groups	Reduced SOD1 concentration in the CSF and NfL levels in the plasma over 28 weeks No improvement of clinical endpoints Associated with adverse events (AEs)
	An antisense oligonucleotide against SOD1 delivered intrathecally for patients with SOD1 familial amyotrophic lateral sclerosis: a phase 1, randomised, first-in-man study [15] Clinicaltrials.gov number, NCT01041222	Phase 1, double-blind, placebo-controlled, dose-escalation study. In this study, four dose levels (0.15, 0.5, 1.5, and 3 mg) of ISIS 333611 are sequentially evaluated. Each dose is administered at a rate of 0.25 mL/12 h. Each dose level is studied in a cohort of 8 patients, with 6 randomly receiving active treatment and 2 receiving placebo	Primary outcomes: safety, tolerability, and pharmacokinetics of the ISIS 333611 at four dose levels (safety analysis of dose escalation after Day 8 of the study) Secondary outcome: NA	When administered intrathecally, the ISIS 333611 has good tolerability with no occurrence of severe AEs
	Study of WVE-004 in Patients With C9orf72-associated Amyotrophic Lateral Sclerosis (ALS) or Frontotemporal Dementia (FTD) (FOCUS-C9) ClinicalTrials.gov number, NCT04931862	Phase 1b/2a multicenter, randomized, double-blind, placebo-controlled study where the experimental group receives intrathecal administration of WVE-004, and the control group receives intrathecal administration of placebo	Primary outcome: Proportion of patients experiencing AEs Secondary outcomes: Pharmacokinetics (concentration of WVE-004 in CSF) and pharmacodynamics (change in poly-GP levels in CSF relative to baseline)	NA (trial not completed)
	Open-label Extension (OLE) Study of WVE-004 in Patients With C9orf72-associated Amyotrophic Lateral Sclerosis (ALS) and/or Frontotemporal Dementia (FTD) ClinicalTrials.gov number, NCT05683860	A multicenter, OLE study in which participants receive intrathecal administration of WVE-004	Primary outcome: Safety (number of patients experiencing AEs; number of patients experiencing serious AEs; number of patients who discontinue due to AEs) Secondary outcomes: NA	NA (trial not completed)
	Study to Assess the Safety, Tolerability, Pharmacokinetics, and Effect on Disease Progression of BIIB078 Administered to Previously Treated Adults C9ORF72-Associated Amyotrophic Lateral Sclerosis (ALS) ClinicalTrials.gov number, NCT04288856	An extension study where BIIB078 will be administered in 3 doses during loading phase, approximately 2 weeks apart, followed by maintenance doses administered approximately every 4 weeks via intrathecal infusion	Primary outcome: Number of participants experiencing AEs Secondary outcomes: Serum concentration of BIIB078; CSF concentration of BIIB078	NA (trial not completed)
	A Study to Assess the Safety, Tolerability, and Pharmacokinetics of BIIB078 in Adults With C9ORF72-Associated Amyotrophic Lateral Sclerosis [226] ClinicalTrials.gov number, NCT03626012	A Phase 1 multiple ascending dose study where BIIB078 is administered in 6 cohorts. The experimental group receives a loading regimen of 3 doses on the first day and subsequently for two more days, followed by 5 maintenance doses over the next five days The control group receives placebo, with different dosing schedules across cohorts	Primary outcomes: number of participants experiencing AEs and serious adverse events (SAEs) Secondary outcomes: serum concentration of BIIB078; area under the concentration–time curve from time 0 to infinity (AUC_inf_); area under the concentration–time curve from time 0 to the last measurable concentration (AUC_last_); maximum observed concentration (*C*_max_); time to reach C_max_ (*T*_max_); terminal elimination half-life (*t*_1/2_); change from baseline in ALSFRS-R score; change from baseline in percent predicted slow vital capacity (SVC); change from baseline in muscle strength; change from baseline in bulbar strength	Compared to the placebo group, patients in the BIIB078 treatment group showed no reduction in neurofilament levels and did not benefit in terms of clinical outcomes
	FUSION: A Study to Evaluate the Efficacy, Safety, Pharmacokinetics and Pharmacodynamics of ION363 in Amyotrophic Lateral Sclerosis Participants With Fused in Sarcoma Mutations (FUS-ALS) ClinicalTrials.gov number, NCT04768972	A multicenter Phase 1–3 study where the experimental group receives ION363 via lumbar intrathecal infusion every 12 weeks. In the first part, there is a 60-week double-blind treatment period with additional loading doses at 4 weeks. The open-label extension (Part 2) continues for 84 weeks with doses every 12 weeks and an additional loading dose 4 weeks after the first dose. In Part 3, patients may continue open-label ION363 every 12 weeks for up to 3 years, until ION363 is approved in their country/region, or until the sponsor terminates the development program, whichever is earlier The control group receives placebo via lumbar IT infusion every 12 weeks with an additional loading dose at 4 weeks during the 60-week double-blind treatment period (Part 1)	Primary outcome: change in functional disability from baseline (Day 1 of Part 1) to day 505 of the study Secondary outcomes: Change from baseline to day 505 of the study in Amyotrophic Lateral Sclerosis Specific Quality of Life—Revised (ALSSQOL-R) score; survival and ventilation-free survival; clinical SVC, HHD, concentration of NfL in CSF, and concentration of FUS in CSF	NA (trial not completed)
	A Study to Assess the Safety, Tolerability, and Effect on Disease Progression of BIIB105 in Participants With Amyotrophic Lateral Sclerosis (ALS) and Participants With the ALS Ataxin-2 (ATXN2) Genetic Mutation (ALSpire) ClinicalTrials.gov number, NCT04494256	A Phase 1/2 multiple ascending dose study with a long-term open-label extension. In the experimental group, during Part 1, participants receive BIIB105 intrathecally as 3 loading doses over the first day and the following two days, followed by two maintenance doses over the subsequent two days. In Part 2, dosing continues according to cohorts The control group receives placebo administered according to cohort schedules	Primary outcome: number of participants experiencing AEs and SAEs Secondary outcomes for different parts: Part 1: Serum and CSF concentration of BIIB105; Area under the concentration–time curve from time 0 to infinity (AUC_inf_) of serum concentration; Area under the concentration–time curve from time 0 to the last measurable concentration (AUC_last_) of serum concentration; etc Part 1 combined with Part 2: Serum concentration of BIIB105; Change from baseline in plasma NfL levels; etc	NA (trial not completed)
RNAi	Safety, Tolerability, and Exploratory Efficacy Study of Intrathecally Administered Gene Therapy AMT-162 in Adult Participants With SOD1 Amyotrophic Lateral Sclerosis (*SOD1*-ALS) ClinicalTrials.gov number, NCT06100276	Phase 1/2 multicenter, single ascending dose study exploring three dose levels of AMT-162, with approximately 6 to 12 participants in total. Each participant receives a single dose of AMT-162 via intrathecal infusion and will undergo follow-up for up to 5 years following administration of AMT-162	Primary outcome: safety and tolerability of intrathecal administration of AMT-162 in participants with *SOD1*-ALS Secondary outcomes: immune responses to AMT-162 and dropout rates following intrathecal administration of AMT-162; efficacy of intrathecal AMT-162, including changes from baseline in percent predicted SVC, HHD scores, and serum levels of NfL protein	NA (trial not completed)
	Safety and Tolerability of RAG-17 in the Treatment of Amyotrophic Lateral Sclerosis Patients With *SOD1* Gene Mutation ClinicalTrials.gov number, NCT06556394	An open-label, single-center, first-in-human dose escalation study where patients initially receive 60 mg of RAG-17. If within 14 days following the first dose there are no AEs or SAEs, patients may proceed to dose escalation every 30 mg every 14 days during an observation period. The study plans to increase doses 3 to 4 times until reaching the dose-limiting toxicity, with the optimal dose used for a continuous 6-month treatment period. Subsequent doses for continuous treatment will be selected between the safe dose and maximum tolerated dose as the optimal continuous treatment dose	Primary outcomes: AEs and SAEs; Clinical laboratory parameters (blood and urine tests); Physiological parameters—vital signs; Revised ALSFRS-R; System-specific examinations (neurological, respiratory, digestive, circulatory); Electrocardiogram results. Secondary outcomes: SOD1 protein levels; Plasma NFL levels; Pharmacokinetic changes of RAG-17; Mechanical ventilation time; Mortality.	NA (trial not completed)
CRISPR-Cas9	NA	NA	NA	NA

### ASOs

ASOs are short synthetic DNA or RNA chains. They are typically designed to be complementary to specific messenger RNA (mRNA) sequences within cells [14]. ASOs work by recruiting Ribonuclease H (RNase H) when binding to target mRNAs [15, 16]. The RNase H can recognize the RNA–DNA or RNA-RNA duplex formed by the ASO and its target mRNA, and then cleave the mRNA strand of the duplex, leading to degradation of the target mRNA. This results in reduced production of the protein encoded by the mRNA [14]. ASOs are used to reduce the production of harmful proteins in certain genetic disorders like ALS [14].

### RNAi

RNAi is a natural cellular process that uses small RNA molecules to silence the expression of specific genes. In the context of ALS, RNAi can reduce the production of proteins that cause the disease. It typically involves molecules such as small interfering RNA (siRNA) or microRNA (miRNA) [17]. These RNA molecules guide the RNA-induced silencing complex (RISC) to complementary mRNA molecules, leading to their degradation or preventing their translation into proteins. siRNA and mRNA are perfectly complementary, leading RISC to directly cleave the target mRNA, resulting in its degradation. miRNA and target mRNA are partially complementary, usually at the 3′ untranslated region (3′ UTR), primarily inhibiting mRNA translation or causing mRNA instability and degradation [18]. RNAi has been exploited therapeutically to silence specific genes involved in disease processes [19].

### CRISPR-mediated gene editing

CRISPR represents the most advanced genetic engineering approach, allowing for precise editing of DNA within cells. It can be used to correct mutations that cause ALS or to introduce new genetic material that can counteract the disease's effects [20]. Mechanistically, CRISPR uses guide RNA (gRNA) to direct the Cas9 enzyme to specific locations in the DNA. Once bound to the target DNA sequence, Cas9 induces a double-strand break in the DNA [20]. The subsequent repair of this break introduces changes in the gene sequence, which result in knockout of harmful genes or correction of genetic mutations [21].

## Gene therapy vectors

Gene vectors are tools or systems used to deliver exogenous genes into target cells or organisms, typically in combination with the aforementioned gene therapy strategies. In the following sections, we will introduce several gene vectors that play important roles or have promising potentials in ALS gene therapy [22, 23]. The characteristics and applications of gene vectors are presented in Table 2.

**Table 2:** Applications of gene therapy vectors

Vector type	Name	Advantages	Disadvantages	Applications of gene therapy in ALS
Viral vectors	Ad	Safe and effective gene vectors in early gene therapy studies	1. Ad can easily trigger strong immune and inflammatory responses in patients 2. The duration of transgene expression is very limited	Ad-GDNF therapy can prevent the loss of motor neurons in a transgenic mouse model with *SOD1* mutations by preserving the survival p-Akt (phosphorylated Akt) signaling without affecting caspase activation [26]
	AAVs	1. Do not elicit significant immune responses or cause disease 2. Effectively infect mammalian cells without the need for helper viruses 3. Extensively studied with broad application prospects	1. Traditional AAVs have low penetration ability through the blood–brain barrier 2. Traditional AAVs have low central nervous system- targeting specificity Some AAVs have high peripheral tissue targeting affinity, leading to off-target effects 3. Novel AAVs with high central nervous system affinity or central administration methods can cause internal CNS diffusion, leading to various central adverse effects 4. Previous exposure to related viruses can result in memory humoral immune elimination of AAV upon subsequent use	1. Subpial delivery mediated by AAV is a currently safer gene therapy technique under development [29, 30] 2. AAV9-SynCav1 delivered via spinal subpial delivery preserves neuro-muscular function and α-motor neurons in the ventral horn of double transgenic (SynCav1 TG/hSOD1^G93A^) mice [29] 3. Subpial delivery of AAV9-shRNA-SOD1 in SOD1^G37R^ mouse models shows extensive gene silencing, significant preservation of motor function and α-motor neurons, and halts disease progression in symptomatic mice [30] 4. AAV-IGF-1 promotes IGF-1 synthesis in spinal motor neurons of ALS mice carrying the hSOD1 mutation, successfully prolonging mouse survival [31] 5. AAV-NDNF, administered early after symptom onset, improves motor performance and weight maintenance; mid-term administration enhances motor abilities, and late-stage injections extend lifespan [32] 6. AAV-mediated CRISPR-Cas9 gene editing demonstrates gene regulation capabilities in mouse cochleae, expanding the potential applications of AAVs in gene therapy, with future opportunities for ALS treatment [34]
	Lentiviral Vectors	Can achieve long-lasting transgene expression at low titers	1. Difficulty in crossing the blood–brain barrier, with gene transduction primarily limited to areas near the injection site 2. Retroviral vectors may integrate DNA into the host chromosome, potentially leading to abnormal expression and tumor formation	1. The use of lentiviral vectors expressing VEGF via retrograde transport at nerve terminals has been shown to protect neurons, significantly delaying the progression of ALS in SOD1^G93A^ mutant mice [38] 2. Lentiviral vectors carrying shRNA, delivered to primary neuronal cells, silence *SOD1* expression, ultimately delaying the onset of symptoms and extending survival in *SOD1*-ALS mice [17]
Non-viral vectors	Liposomes	1. Biocompatible, allowing delivery of lipophilic or hydrophilic therapeutic agents, effectively penetrating the blood–brain barrier for targeting the central nervous system 2. Novel lipid nanoparticles are smaller than traditional ones, exhibit higher in vivo stability, greater therapeutic payload capacity, lower immunogenicity, enhanced nucleic acid encapsulation, and improved in vivo release capabilities	1. Short duration in vivo, stability needs improvement 2. High precision required for liposome formulations; incompatibility with drugs can lead to drug leakage or ineffectiveness	1. Liposomes as carriers for transporting riluzole and verapamil effectively penetrate the central nervous system for ALS treatment [43] 2. CaP lipid nanoparticles efficiently and safely deliver ASOs, reducing levels of mutant SOD1 in motor neurons of ALS mice [45]
	Naked DNA/RNA Vectors	1. Simple and straightforward use, with strong controllability 2. Relatively simple and convenient preparation and purification processes 3. Low immunogenicity, avoiding carrier-related side effects	1. Low transfection efficiency 2. Insufficient selectivity for specific cells 3. Poor stability in vivo	The gene encoding TTC was cloned into the pcDNA eukaryotic expression vector and intramuscularly injected into transgenic SOD1^G93A^ mouse models, significantly delaying symptom onset and functional deficits while improving the survival rate of spinal motor neurons [46]
	Protein Vectors	1. High specificity of targeting, strong cellular penetration capability 2. Excellent drug release regulation	1. Complex preparation, limited drug loading capacity 2. Potential immunogenicity and biocompatibility issues	A protein gene therapy vector targeting TDP-43 was designed with high affinity to 14–3-3θ, effectively alleviating functional deficits and neurodegeneration in TDP-43 mutant ALS/FTD mouse models [47]
	Exosomes	1. Natural carriers with good immunogenicity and biocompatibility 2. Strong cell targeting and high stability in vivo	1. Difficult production and purification 2. Complexity of components and functions, which may lead to various adverse effects	Extracellular vesicles derived from stem cells targeting the optimization of the tryptophan-melatonin pathway in cells can be utilized for treating early-stage ALS [49]

### Viral Vectors

Viral vectors play a crucial role in ALS gene therapy. They can effectively deliver therapeutic genes or gene-editing tools to patients' cells. Below are several viral vectors commonly used in ALS gene therapy.

#### Adenovirus

Replication-deficient adenoviruses are safe and effective gene delivery vectors used in early gene therapy research. They have the ability to express various genes in multiple organs (such as the liver, lungs, and muscles) and the central nervous system (CNS) [24]. Haase et al. demonstrated that adenovirus-mediated *NT-3* (neurotrophin-3) gene transfer by intramuscular injection extended the lifespan, reduced the loss of motor axons, and improved neuromuscular function of mice with progressive motor neuronopathy [25]. Manabe et al. investigated the therapeutic effects of adenovirus-mediated glial cell line-derived neurotrophic factor (*GDNF*) gene transfer in a SOD1 mutant transgenic (Tg) mouse model. The adenovirus-*GDNF* treatment prevented motor neuron loss by preserving the phosphorylated Akt (p-Akt) signaling without affecting caspase activation [26]. These studies were significant at the time for guiding the treatment of ALS and other motor neuron diseases.

However, further research revealed that adenovirus vectors not only induce strong immune and inflammatory responses in patients but also have limited duration of transgene expression [27]. Consequently, current viral vector research has gradually shifted towards other safer and more durable vectors.

#### AAV vectors

AAV is a non-pathogenic, small virus belonging to the single-stranded DNA virus family. Compared to adenoviruses, AAV does not elicit significant immune responses or diseases and can effectively infect mammalian cells without helper viruses. Numerous studies have highlighted the significance of AAV-mediated gene therapy in ALS [28]. AAV-mediated subpial delivery is relatively safe. Wang et al. showed that subpial delivery of AAV9-synapsin-caveolin-1 (AAV9-*SynCav1*) preserved neuromuscular function and α-motor neurons in the ventral horn of SynCav1 TG/hSOD1^G93A^ double transgenic mice [29]. Hernandez et al. demonstrated promising results using subpial delivery of AAV9-shRNA-*SOD1* to treat ALS in SOD1^G37R^ mice. The study involved both pre-symptomatic and symptomatic stages of injection. Results showed extensive gene silencing, significant preservation of motor function and α-motor neurons, and blocking of disease progression in symptomatic mice. These findings were replicated in large animals, indicating potential for human application. The research suggests that early intervention using this method could effectively treat ALS, paving the way for human clinical trials [30].

Meanwhile, previous ALS experiments primarily employed adenovirus vectors to mediate the delivery of neurotrophic factors. Recently, similar experiments have been increasingly conducted using AAV vectors. Over 20 years ago, Kaspar et al. used an AAV encoding the neurotrophic factor insulin-like growth factor 1 (IGF-1) to promote the synthesis of IGF-1 within the spinal motor neurons of ALS mice carrying the human SOD1 (hSOD1) mutant gene, successfully extending the survival of these mice [31]. In recent years, a study emphasized the therapeutic potential of AAV-mediated neuron-derived neurotrophic factor (NDNF) in SOD1^G93A^ mouse models. This study utilized the AAV-PHP.eB capsid for widespread expression in the brain and spinal cord. Administration of AAV-NDNF shortly after symptom onset improved motor performance and weight maintenance; mid-stage administration still enhanced motor abilities; and late-stage injection extended lifespan. NDNF promoted spinal motor neuron survival, reduced protein aggregation, preserved neuromuscular function, activated survival pathways, and reduced apoptosis. This approach presents a promising ALS treatment strategy [32].

With continuous advancements in AAV transgene technology, AAV-mediated gene delivery has become a key method in gene therapy, particularly in the context of ALS research, including its application in C9-ALS human induced pluripotent stem cell (iPSC) models. This strategy involves delivering genetic material to cells to mitigate the effects of harmful gene mutations. Furthermore, Wang et al. demonstrated that the AAV9-mediated gene delivery could elevate the levels of neuroprotective proteins in ALS mouse models, indicating therapeutic benefits [29]. Cappella et al. discussed the potential of iPSCs as models for developing and testing gene therapies, including AAV-mediated strategies for neuromuscular and motor neuron diseases [33]. Additionally, Kang et al. expanded the potential applications of AAV in gene therapy by demonstrating the efficacy of AAV-mediated CRISPR-Cas9 gene editing in the inner ears of mice [34]. Finally, Depla et al. discussed the use of iPSC-derived brain organoids as models for selecting and testing recombinant AAV (rAAV) capsids for AAV-based gene therapy, highlighting the potential to enhance translational research in gene therapy [35]. Overall, these studies underscore the versatility and efficacy of AAV-mediated gene delivery in ALS research while also emphasizing the complexities and challenges associated with this therapeutic approach.

Previously, the main issues with AAV vectors included low permeability through the blood–brain barrier (BBB), low specificity of CNS targeting, and high affinity for peripheral tissues (e.g., the liver). Although recent advancements in AAV vectors that do not specifically bind to peripheral organs (e.g., AAV.cap-10) and CNS administration methods have largely addressed these issues, adverse consequences resulting from AAV diffusion within the CNS still require further investigation. Additionally, the elimination of AAV by memory humoral immunity due to prior viral exposure is also a problem that needs further exploration [27, 36].

#### Lentivirus vectors

Lentiviral vectors are a type of retroviral vector that can achieve sustained transgene expression at low titers [37]. In a study by Azzouz et al., lentiviral vectors expressing vascular endothelial growth factor were retrogradely transported via nerve terminals, exerting neuroprotective effects and significantly delaying the progression of ALS in *SOD1* (G93A) mutant mice [38]. Similarly, Ralph et al. demonstrated that the lentiviral-mediated silencing of *SOD1* using short-hairpin RNA (shRNA) significantly delayed the onset of symptoms and extended survival in SOD1-ALS mice [17].

However, lentiviral vectors also present certain challenges. Compared to AAV vectors, lentiviral vectors have a lower ability to cross the BBB, and their gene transduction is primarily confined to areas near the injection site [39]. Recent studies suggest that dual targeting with CS-TeTIM (core streptavidin fused with full-length nontoxic tetanus toxin) combined with rabies glycoprotein can effectively enhance the retrograde transduction of lentiviral vectors in ALS motor neurons in vivo [40]. However, the integration of retrovirus-derived DNA into the host chromosome, potentially leading to tumorigenesis, limits the application of this type of retroviral vector [39].

### Non-viral vectors

Non-viral vectors play a significant role in gene therapy, offering a relatively safe and effective method for delivering genetic material to target cells while minimizing the potential risks associated with viral vectors, such as immune response and genetic mutations. Here are some commonly used non-viral vectors in ALS gene therapy.

#### Liposomes

Liposomes are common carriers for gene and drug delivery. Composed of a phospholipid bilayer and an aqueous core, liposomes are biocompatible and can deliver both lipophilic and hydrophilic therapeutic agents, effectively crossing the BBB to target the CNS [41, 42]. Riluzole, the currently approved drug for ALS, is also a substrate for P-glycoprotein (P-gp), an efflux transporter at the BBB, which reduces its efficacy. Yang et al. used liposomes to co-deliver riluzole and verapamil, a P-gp inhibitor, enhancing riluzole transport to the CNS for ALS treatment [43]. Similar studies combining liposomes with effective ALS-targeted drugs are ongoing, though their application in gene therapy remains limited.

With advancements in nanotechnology, lipid nanoparticles (LNPs) offer advantages over traditional liposomes, including their smaller size, higher in vivo stability, greater therapeutic loading capacity, lower immunogenicity, and more efficient nucleic acid encapsulation and release [44]. Ediriweera et al. have demonstrated the use of calcium phosphate LNPs as carriers to effectively and safely deliver ASOs and reduce mutant SOD1 levels in mouse motor neurons [45]. This indicates the potential for nanoparticles to advance the application of non-viral vectors in ALS gene therapy.

#### Naked DNA/RNA vectors

Naked DNA/RNA delivery is a novel, safe gene therapy strategy. María et al. cloned the gene encoding the tetanus toxin heavy chain (TTC) into the pcDNA eukaryotic expression plasmid and injected it intramuscularly to transgenic SOD1^G93A^ mice. The TTC-targeted therapy significantly delayed the onset of symptoms and functional deficits and increased spinal motor neuron survival [46].

Although naked DNA transfection is a relatively simple, non-viral gene delivery method, it faces challenges such as low transfection efficiency and lack of cell-specific targeting, limiting its application in recent studies.

#### Protein carriers

Protein carriers also play a crucial role in ALS gene therapy by facilitating gene delivery, regulating gene expression, and enabling targeted delivery. The 14-3-3θ protein, a member of the 14-3-3 protein family, is widely present in cells and involved in regulation of signal transduction, cell cycle control, and apoptosis. Ke et al. designed a gene therapy vector targeting TAR DNA-binding protein (TDP-43) pathology based on the high affinity between 14-3-3θ and the pathogenic TDP-43, significantly alleviating functional deficits and neurodegeneration in different TDP-43 mutant ALS/FTD mouse models [47].

Although initial experimental results are promising, more research is needed to further confirm the efficacy and safety of protein carriers as novel gene therapy vectors in the ALS field.

#### Exosomes

Mesenchymal stromal cells (MSCs) have gained attention for treating neurodegenerative diseases. Exosomes derived from MSCs offer a novel cell-free therapeutic strategy. Bonafede et al. demonstrated that exosomes derived from adipose tissue-derived MSCs, containing diverse protein contents and complex nucleic acid components, exert significant neuroprotective effects in an in vitro ALS model [48]. Anderson proposes that stem cell-derived exosomes can be used to optimize the tryptophan-melatonin pathway in cells for early ALS treatment, through exosomal miRNAs and 14-3-3 isoforms. When applied to muscles, this approach could optimize muscle release of N-acetylserotonin and brain-derived neurotrophic factor (BDNF), exerting protective effects in early ALS [49].

Exosomes can deliver molecular drugs to damaged CNS areas and promote recovery. This property suggests that combining exosomes as gene therapy vectors with drugs is a potential treatment approach for ALS and other neurodegenerative diseases [50].

## Targets of gene therapy

### Classical targets

#### SOD1

In the field of ALS gene therapy, SOD1 has emerged as a crucial target due to its significant role in the disease mechanism. Research has elucidated that *SOD1* mutations lead to ALS through toxic gain-of-function mechanisms, primarily via misfolded SOD1 aggregates [51]. Recent studies have identified that both mutant and wild-type SOD1, as well as TDP-43, exhibit propagated protein misfolding properties, potentially underpinning the observed spread of the disease along the neuroaxis [52]. Aggregation of hSOD1 plays a pivotal role in ALS pathogenesis. The electrostatic loop of hSOD1 containing charged residues is crucial for guiding negatively charged superoxide substrates toward the Cu^2+^-centered active site. The structure and charge distribution of this loop significantly contribute to the catalytic properties and enzymatic activity of hSOD1 [53]. Transmission electron microscopy has shown that two ALS-related mutations of *SOD1* (G138E and T137R) promote the formation of amyloid-like SOD1 aggregates. Studies indicate enhanced amyloid formation under destabilizing environment (e.g., altering the charge distribution of SOD1 through mutations) in ALS mutants compared to wild-type SOD1 [53]. Additionally, *SOD1* mutations can lead to oxidative damage. Studies on *SOD1*-ALS patients and models have shown varying degrees of DNA damage, suggesting their role in motor neuron degeneration [54]. These mutations, involving over 187 variants, account for 12%–20% of familial ALS cases and 1%–2% of sporadic ALS cases. *SOD1* mutations are also associated with clinical variability [55]. Studies by Ghadge et al. and Tokuda et al. have focused on the therapeutic potential of targeting misfolded SOD1 in mutant and wild-type forms, respectively [56, 57]. In addition, Gidalevitz et al. reported that the specific toxic phenotypes of *SOD1* mutations are defined by their genetic interactions with temperature-sensitive mutations of other genes in *C. elegans* [58].

SOD1 has appeared as a promising target of gene therapy strategy for ALS treatment. Yang et al. reported that hSOD1^G93A^ cell and mouse models show activation of ferroptosis, accompanied by decreased nuclear retention of nuclear factor erythroid 2-related factor 2 (NRF2). RTA-408, an activator of NRF2, reduces ferroptosis in hSOD1^G93A^ NSC-34 cells and improves motor function of hSOD1^G93A^ ALS mice, suggesting NRF2 as a potential therapeutic target for ALS. While insightful, the study highlights the need for further research, particularly regarding different ALS mutations [59].

McCampbell et al. used next-generation ASOs targeting *SOD1* for ALS treatment. These ASOs show enhanced efficacy compared to an early-generation ASO, significantly reducing SOD1 mRNA and protein levels in SOD1^G93A^ rats and mice. Notably, the *SOD1* ASO extended survival by over 50 days in rats and by nearly 40 days in mice. Moreover, the loss of muscle action potentials and increases of serum phosphorylated neurofilament levels were reversed by the *SOD1* ASO, indicating a potential reversal of ALS symptoms. These results provide a solid foundation for advancing these ASOs into human clinical trials, highlighting their prospects in ALS treatment [60].

Tofersen (Qalsody™), an ASO targeting *SOD1* mRNA, was approved in the United States on April 25, 2023, for treating ALS in adults with *SOD1* gene mutations. This accelerated approval was based on reductions in plasma neurofilament light chain (NfL) levels, a biomarker for neurodegenerative diseases. The Phase III VALOR trial of Tofersen, which is part of a three-part study including Phase I/II trials, demonstrated reductions in cerebrospinal fluid (CSF) SOD1 and plasma NfL levels, although it did not significantly improve clinical endpoints. The recommended dose of tofersen for intrathecal administration is 100 mg every 28 days following an initial loading dose. Most adverse events were mild or moderate, related to ALS progression or lumbar puncture. However, severe adverse events, such as meningitis and aseptic meningitis, were observed in 7% of recipients. The approval of tofersen depends on further confirmation of clinical benefits. Tofersen is now under review in the EU. Phase III development is ongoing globally [61].

Miller et al. assessed the safety and pharmacokinetics of tofersen (an ASO targeting *SOD1* mRNA) in adults with ALS caused by *SOD1* mutations. In this escalating-dose trial, 50 participants were randomized to receive intrathecal tofersen or placebo over 12 weeks. On day 85, CSF SOD1 concentrations were significantly reduced, particularly at the highest dose. Common adverse events included headache, procedural pain, and post-lumbar puncture syndrome. Severe adverse events occurred in both tofersen and placebo groups, including three deaths related to ALS progression or complications. The study underscores the potential of tofersen in reducing SOD1 protein synthesis in ALS patients and emphasizes the need for careful safety monitoring [62].

A Phase III clinical trial conducted from March 2019 to July 2021, evaluated the efficacy and safety of tofersen in adults with ALS associated with *SOD1* mutations. In this trial, subjects were randomized to receive tofersen or placebo, focusing on changes in ALS Functional Rating Scale-Revised (ALSFRS-R) scores as well as CSF SOD1 and plasma NfL levels. Results showed significant reductions in SOD1 and NfL levels, although no significant differences were observed in ALSFRS-R scores or secondary clinical endpoints between the tofersen and placebo groups. Open-label extension involved 88% of participants, with 7% of the tofersen recipients experiencing notable adverse events, including meningitis and aseptic meningitis. The study highlights the potential of ASO therapy for ALS treatment while stressing the need for further research to understand its clinical efficacy [63].

#### C9orf72

The expanded GGGGCC hexanucleotide repeat (HRE) in the *C9orf72* gene is the most common genetic cause of familial ALS and frontotemporal dementia (FTD). *C9orf72* plays an important role in triggering toxic gain-of-function mechanisms leading to neurodegeneration [64, 65]. Therapeutic advances targeting the pathological expansion of *C9orf72* through innovative strategies, including ASOs and RNA interference, offer new hope for addressing this challenging genetic factor [66]. Research by Donnelly et al. has demonstrated the potential of antisense interventions in mitigating RNA toxicity, marking a significant step toward combating these neurodegenerative diseases [67].

**Fig. 2:**
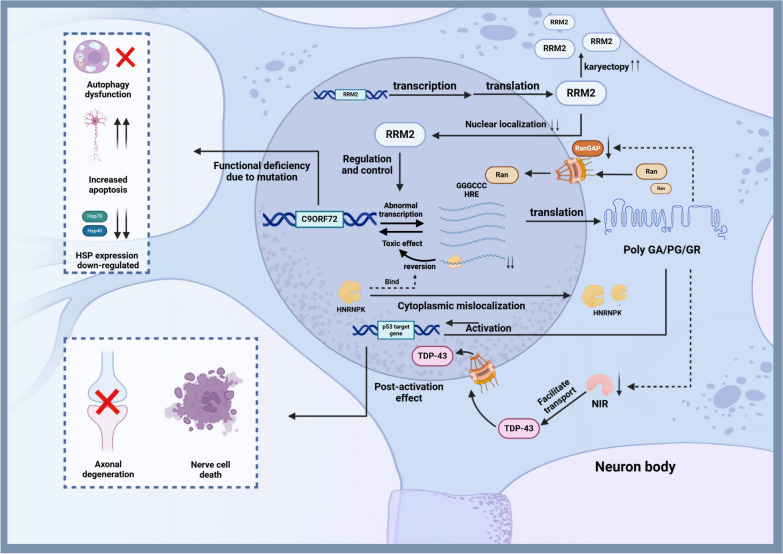
Pathogenesis of *C9orf72* mutations. The GGGGCC HRE in the *C9orf72* gene leads to production of toxic RNA. The RNA-binding protein HNRNPK, which can reverse the toxicity of sense and antisense repeat RNAs, undergoes mislocalization in the cytoplasm. RRM2, a downstream target of HNRNPK involved in DNA damage response, exhibits increased nuclear translocation but decreased expression in ALS.The GGGGCC HRE also leads to production of toxic proteins, including poly-GA, poly-GP, and to a lesser extent, poly-GR DPRs. These toxic proteins activate p53 target genes, exacerbating axonal degeneration and cell death. These proteins can also impair nucleocytoplasmic transport of proteins including TDP-43, by disrupting the function of RanGAP and NIR. Reduced expression of the *C9orf72* gene leads to axonal transport defects, increased apoptosis, downregulation of HSP70 and HSP40, and dysfunction of the autophagy-lysosome pathway leading to accumulation of toxic proteins. Figure created with BioRender.com

Evidence suggests that in C9orf72 ALS/FTD, functional deficits of the C9orf72 protein alone are not sufficient to cause neurodegeneration. Instead, multiple mechanisms are involved [68]. In the following, we will review *C9orf72*-associated mechanisms and potential gene therapy targets (Fig. 2).

From the perspective of abnormal *C9orf72* expression, Masin et al. found that knocking out *C9orf72* in motor neurons with HRE exacerbates axonal transport defects, increases apoptosis, and reduces levels of heat shock protein (HSP)70 and HSP40. In addition, inhibiting these HSPs further worsens the ALS phenotype in motor neurons with HRE. Thus, HRE in *C9orf72* induces ALS pathogenesis through both gain-of-function and loss-of-function mechanisms. Although the mechanisms underlying HRE toxicity remain unclear, the toxicity may result from transcription of HRE RNA or translation of repeat-associated non-AUG (RAN) proteins, leading to toxic gain-of-function, compounded by reduced C9orf72 expression, contributing to ALS and FTD pathogenesis [69]. The abnormal expansion of C9orf72 repeats is closely linked to disruptions of nucleocytoplasmic transport [70]. Research by Sarah et al. demonstrated that the dipeptide repeat proteins (DPRs) translated from the HRE RNA not only cause structural damage to the nucleus and nuclear membrane, but also result in the mislocalization of TDP-43 to the cytoplasm [71]. Nicholas et al. utilized CRISPR-Cas9 to screen for modifiers of DPR toxicity, which offers a new avenue for applying CRISPR-Cas9 in the gene therapy of ALS [21]. The disruption of nucleocytoplasmic transport is closely tied to RanGAP1 and nuclear import receptors (NIRs). The *Drosophila* ortholog of RanGAP1, RanGAP, is a potent suppressor of *C9orf72* HRE-mediated toxicity in flies. However, the accumulation of G4C2 HRE sequesters RanGAP, disrupts its function and impairs its nucleocytoplasmic transport [70]. More recently, studies by Hutten and Hayes et al. revealed that arginine-rich DPRs directly interact with various NIRs, such as Imp⍺, Kapβ1, and Kapβ2, further disrupting the nucleocytoplasmic transport [72, 73]. Interestingly, this chaperone-like characteristic of NIRs makes them a promising therapeutic strategy for neurodegenerative diseases. Enhancing either specific or global NIR expression in gene therapy warrants further investigation [74].

Dysfunction in the autophagy-lysosome pathway, coupled with the toxic effects of C9orf72 repeat RNA and DPRs, drives disease pathogenesis [75]. Boivin et al. found that sense and antisense repeat sequences are translated upon initiation at typical AUG or near-cognate start codons, generating polyGA, polyPG, and less prominently, polyGR-DPR proteins. However, autophagy can prevent the accumulation of these proteins. In addition, reduced expression of the autophagy regulator C9orf72 protein leads to impaired autophagy function, affecting DPR protein clearance, resulting in their toxic accumulation and eventual neuronal cell death. Pharmacological activation of autophagy prevents neuronal cell death caused by DPR protein accumulation. These findings suggest a dual-hit pathogenic mechanism in ALS/FTD, wherein reduced C9orf72 expression synergizes with DPR protein accumulation and toxicity [76]. Additionally, Ciura et al. found that C9orf72 is present as a complex with SMCR8 (Smith-Magenis Syndrome Chromosome Region Candidate Gene 8) and WDR41 (WD Repeat Domain 41). This complex acts as a GDP/GTP exchange factor for Ras-Related Protein Rab-8A (RAB8) and Ras-Related Protein Rab-39B (RAB39), two RAB GTPases involved in macroautophagy/autophagy. C9orf72 also interacts with autophagy receptors optineurin (OPTN) and sequestosome 1 (SQSTM1), potentially mediated through RAB8, RAB39, or other unidentified proteins. Mutations in *OPTN* and *SQSTM1*, in turn, lead to ALS-FTD. SQSTM1-positive aggregates can be observed in ALS-FTD patients with GGGGCC repeat expansion in *C9orf72* [77]. Therefore, further investigation into the connection between autophagy and C9orf72 is warranted.

From the perspective of toxic RNA, the RNA-binding protein heterogeneous nuclear ribonucleoprotein K (HNRNPK) can reverse the toxicity of sense and antisense repeat C9orf72 RNA. This effect depends on the subcellular localization of HNRNPK and RNA recognition, rather than C9orf72 repeat RNA binding. HNRNPK shows cytoplasmic mislocalization in *C9orf72* ALS patients, which is in line with its dysfunction in* C9orf72* ALS. RRM2 (ribonucleotide reductase regulatory subunit M2), a downstream target of HNRNPK involved in DNA damage response, exhibits increased nuclear translocation but decreased expression in C9orf72 ALS/FTD patient tissue. Importantly, increasing HNRNPK or RRM2 expression is sufficient to alleviate DNA damage in the *C9orf72* RNA toxicity zebrafish model. This reinforces RNA toxicity as a pathogenic mechanism in C9orf72 ALS and demonstrates its association with abnormal DNA damage response, opening new therapeutic avenues for C9orf72 ALS/FTD [78].

In the context of RNAi-based gene therapy for *C9orf72*-associated ALS and FTD, extensive research has been conducted to understand and mitigate the inherent RNA toxicity. Key to this therapeutic approach is the use of molecular tools designed to specifically target and silence RNA sequences responsible for disease pathology. For instance, Ralph et al. found that injection of a lentiviral vector expressing RNAi molecules specifically targeting human *SOD1* in various muscle groups of mice can reduce the expression of mutant SOD1 and improve the survival rate of vulnerable motor neurons in the brainstem and spinal cord [17]. Raygene et al. found that RNAi-based gene therapy (delivering AAV5-miC to different types of neuronal cells) can reduce the accumulation of C9orf72 transcripts containing repeat sequences and alleviate ALS pathology in a mouse model [79]. Furthermore, Ortega et al. identified critical proteins involved in RNA metabolism, such as eRF1 (eukaryotic release factor 1), as potential therapeutic targets for mitigating C9orf72 toxicity [80]. These and other studies highlight the potential of RNAi-based gene therapy as a critical strategy targeting specific molecular mechanisms underlying C9orf72-related neurodegenerative diseases.

CRISPR-Cas9 technology is increasingly recognized as a transformative tool for addressing the genetic bases of ALS, particularly the *C9orf72* gene. Krishnan et al. demonstrated that CRISPR-Cas9 can be used to delete the *C9orf72* promoter in motor neurons from ALS/FTD patients, abolishing the production of DPRs and rescuing neurodegeneration associated with ALS [81]. Additionally, other studies have utilized CRISPR-mediated techniques to lower the expression of C9orf72 variants containing repeat expansions [82]. These findings underscore the potential of the CRISPR technology as a powerful approach to addressing key pathological features of ALS and related neurodegenerative diseases.

ASOs have emerged as a critical focus in ALS research, particularly in targeting transcripts of *C9orf72*. This approach has shown efficacy in reducing nuclear RNA foci associated with C9orf72 in ALS, reversing abnormal gene expression, and decreasing excitotoxicity in iPSC-derived neurons. Notably, gapmer ASOs targeting *C9orf72* repeat expansion transcripts lead to recovery of disease-associated phenotypes in patient-derived fibroblasts [83]. Additionally, in motor neurons of the spinal cord, variant-selective stereopure ASOs significantly decreased sense RNA foci and DPR proteins without disrupting protein expression, and prevented pathology associated with *C9orf72* repeat expansions, highlighting promising avenues for ALS treatment [84].

Finally, the interaction between p53 and C9orf72 cannot be overlooked. RNA sequencing (RNA-seq) revealed specific activation of p53 target genes in mouse primary cortical neurons expressing a 50-repeat poly(PR) protein, (PR)_50_. These genes include *Cdkn1a*, *Puma* (Bbc3), *Trp53inp1*, *Ccng1*, *Sulf2*, and *Fam212b*, indicating a crucial role for p53 in mediating neuronal responses to *C9orf72* mutations [85]. Neuronal knockout (KO) of p53 fully protected neurons from (PR)_50_-induced degeneration, extending to axonopathy and cell death. p53 ablation also inhibited axonopathy induced by poly(glycine-arginine) (GR)_50_, which is another toxic dipeptide repeat sequence produced from *C9orf72* repeat expansions [85]. DNA damage observed in neurons treated with PR50 and iPSC-derived motor neurons from *C9orf72* ALS patients is mediated by p53, indicating a complex mechanism activated by C9orf72 mutations that elicit p53 responses [85].

### *TARDBP*

**Fig. 3:**
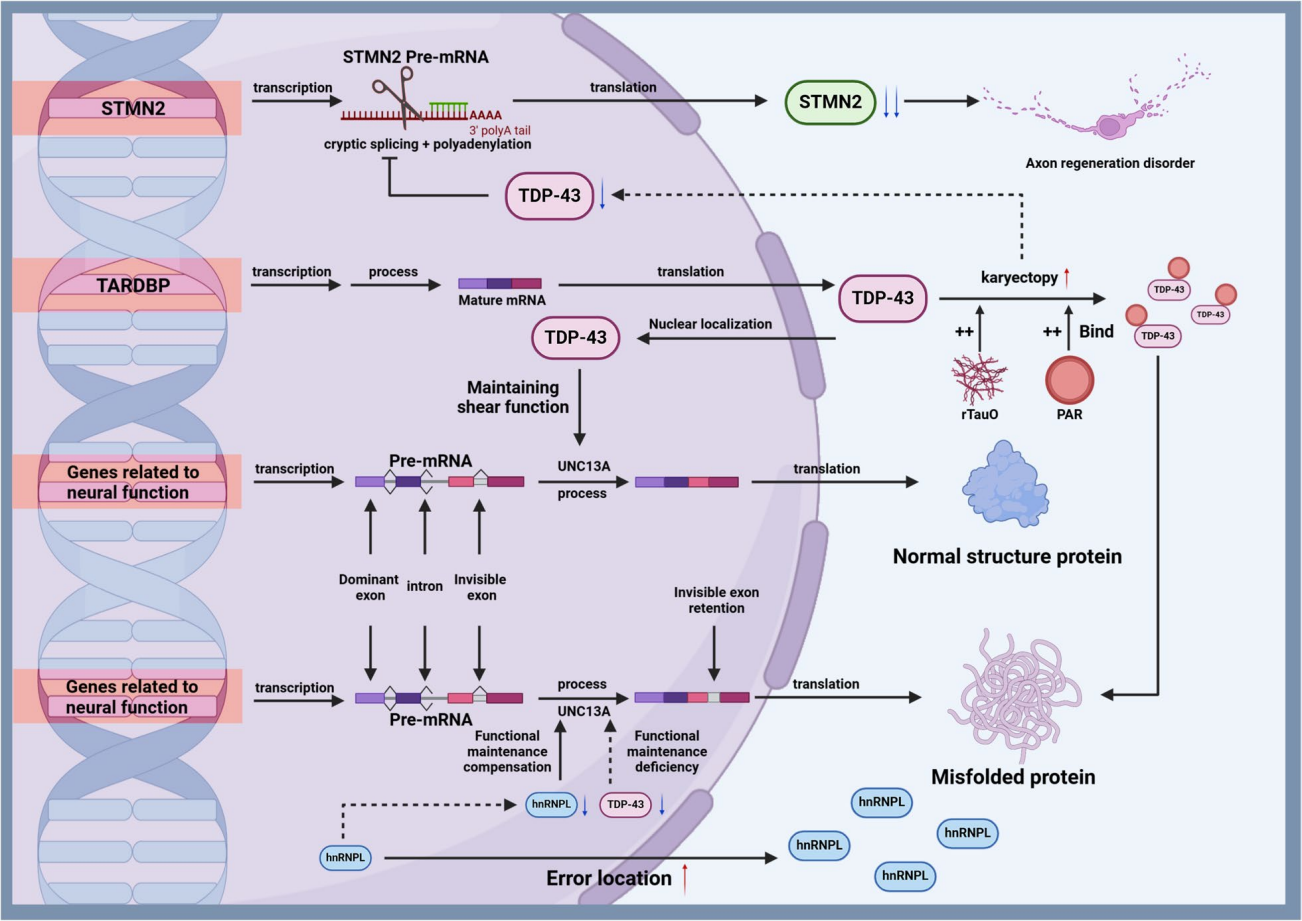
Pathogenesis of *TARDBP* gene mutations. TDP-43 plays a crucial role in RNA/DNA binding and RNA metabolism, and its misfolding leads to the formation of toxic inclusions. The mislocalization and aggregation of TDP-43 trigger DNA damage responses and impair DNA repair mechanisms. Stress granules enriched in PAR bind to key proteins such as TDP-43 and FUS, resulting in abnormal localization and aggregation. Additionally, rTauO affects the localization and aggregation of TDP-43, promoting its translocation from the nucleus to the cytoplasm. TDP-43 mutations disrupt the mRNA splicing regulatory function of UNC13A, causing the inclusion of cryptic exons during RNA splicing and the production of abnormal proteins. In the absence of TDP-43 or in the presence of its mutations, hnRNP L acts as a disease modifier by binding to UNC13A RNA and inhibiting cryptic exon inclusion. Mislocalization of hnRNPs further contributes to neurodegeneration. Furthermore, the loss of TDP-43’s protective function over *STMN2* pre-mRNA results in cryptic splicing and polyadenylation, leading to reduced STMN2 expression and impairing the axonal regeneration capacity of motor neurons. Figure created with BioRender.com

The *TARDBP* gene, which encodes the TDP-43 protein, has significant involvement in the pathogenesis of ALS. TDP-43 is crucial for RNA/DNA binding and RNA metabolism. TDP-43 misfolding leads to the formation of pathogenic inclusions [86]. TDP-43 is associated with many ALS cases and plays a role in DNA damage response (DDR) and DNA repair. Mutations or mislocalization of TDP-43 may lead to increased DNA damage due to impaired DDR signaling or DNA repair [54]. *TARDBP* mutations are found in approximately 4% of familial ALS cases and less than 1% of sporadic ALS cases, and are associated with toxic gain of function and increased apoptosis [87]. Further research has demonstrated alterations in RNA splicing and TDP-43 misfolding caused by *TARDBP* mutations. The pathogenic mechanisms of *TARDBP* mutations and potential gene therapy targets are presented in Fig. 3.

TDP-43 is a major pathological protein in most ALS cases and approximately half of FTD cases. It functions to repress the inclusion of cryptic exons during RNA splicing [88]. Unc-13 Homolog A (UNC13A) plays a critical role in synaptic plasticity by regulating synaptic vesicle release and synaptic transmission. UNC13A dysfunction potentially leads to various neurological disorders. Ma et al. using a mini-gene reporter construct demonstrated that when TDP-43 is depleted, risk variants in *UNC13A* exhibit significant levels of selective splicing, with a notable increase in cryptic exon inclusion. This inclusion is not observed in normal neuronal nuclei, indicating that TDP-43 plays a role in maintaining proper splicing of *UNC13A* [89]. This finding directly links genetic variations in *UNC13A* with increased susceptibility to ALS and FTD through splicing alterations [89]. Brown et al. found that TDP-43 depletion further leads to nonsense-mediated decay and loss of UNC13A protein. Moreover, two common intronic polymorphisms in *UNC13A*, closely associated with the risk of ALS and FTD, overlap with TDP-43-binding sites. These polymorphisms enhance the inclusion of cryptic exons in *UNC13A* both in cultured cells and in the brains and spinal cords of patients with ALS/FTD [90]. These results revealed the mechanism by which *UNC13A* variants exacerbate the effects of decreased TDP-43 function, and provide a promising therapeutic target for TDP-43 proteinopathies [90]. It is noteworthy that, at the chromosomal level, genetic variations on chromosome 19 are closely associated with ALS and FTD. Ma et al. and Brown et al. demonstrated that this association is driven by variations in *UNC13A*, with the underlying mechanism involving abnormal TDP-43 localization, a known pathological feature of both diseases [91].

TDP-43 deficiency and dysfunction are also associated with abnormal expression of Stathmin-2 (STMN2). STMN2 is a microtubule regulator involved in normal axonal growth and regeneration. Decreased STMN2 levels have been detected in the post-mortem spinal cords of ALS patients [92]. Studies by Melamed and Michael et al. revealed that TDP-43 binds to the GU-rich region of *STMN2* pre-mRNA to block cryptic splicing and polyadenylation, and depletion or pathogenic mutations of TDP-43 inhibit normal expression of STMN2 [93, 94]. In fact, abnormal expression of STMN2 plays a crucial role in ALS pathogenesis. In a study by Kelsey et al., *STMN2*-knockout mice exhibited impaired motor behavior and distal neuromuscular junction denervation in fast-fatigable motor units, a hallmark of early ALS pathology [95]. Therefore, early intervention targeting STMN2 may represent a promising gene therapy strategy. Recently, CRISPR effector RfxCas13d (CasRx) and ASOs identified through synthetic screening, blocked the misprocessing of *STMN2* pre-mRNA, and restored axonal regeneration of TDP-43-deficient human motor neurons [93].

Besides TDP-43, research has found that heterogeneous nuclear ribonucleoprotein (hnRNP) L, hnRNP A1, and hnRNP A2B1 can also bind to *UNC13A* RNA and repress cryptic exon inclusion, independent of TDP-43 [88]. This implies that hnRNP L may compensate for the loss of TDP-43 function in regulating *UNC13A* splicing [88]. In addition, the hnRNP network binds to and regulates *UNC13A* RNA regardless of the presence of TDP-43 [88]. Alexander et al. have reviewed several unifying mechanisms by which hnRNPs are directly or indirectly related to the pathogenesis of FTD/ALS, including their binding to pathological inclusions and regulation of pre-mRNA splicing. They propose that hnRNPs initiate and propagate neurodegeneration in multiple aspects, such as the vicious cycle of abnormal RNA metabolism, DNA damage, and protein homeostasis dysfunction, with mislocalization of hnRNPs playing a significant role in this process [96]. Therefore, hnRNPs might serve as potential targeting molecules or regulatory factors in gene therapy, modulating disease progression by directly regulating RNA splicing or interacting with other molecules. However, further research is needed to confirm the causal relationship between the network of abnormal hnRNPs and ALS neurotoxicity.

Poly(ADP-ribose) (PAR) is intricately linked with TDP-43 in ALS and FTD. Post-translational modification of TDP-43 and FUS by addition of PAR lead to their abnormal localization and aggregation, contributing to the pathology of neurodegenerative diseases. Inhibiting the activity of PARP-1/2 (PAR polymerase) has shown benefits in various models of ALS and FTD [97]. PAR-enriched stress granules (non-membrane bound, RNA-rich organelles) play a role in the pathology of ALS/FTD. PAR-mediated recruitment of TDP-43 to stress granules prevents the disease-associated phosphorylation and aggregation of this protein. However, chronic stress can lead to the dissolution of these granules and the formation of neurotoxic aggregates [97]. Therefore, targeting PAR-related genes warrants further investigation in future therapeutic research.

Mauro et al. have explored the interaction between TDP-43 oligomers and tau, another key protein involved in ALS and FTD. They utilized an internally developed anti-TDP43O antibody to investigate the seeding properties of TDP-43 oligomers isolated from AD, ALS, and FTD brain tissues [98]. In HEK-293 cells, treatment with recombinant tau oligomers (rTauO) affects the intracellular localization and aggregation of TDP-43 [98]. In human AD brains, TDP-43 oligomers exhibit strong colocalization with β-amyloid aggregates [98]. TDP-43 can act as a template to trigger tau protein aggregation in vitro. This finding suggests a complex interplay between TDP-43 and tau in the pathogenesis of neurodegenerative diseases, although the exact mechanisms and implications of these interactions require further investigation [98].

Recent advances in ALS gene therapy, particularly targeting the *TARDBP* gene, have highlighted the potential of antisense RNAi and CRISPR/Cas9 method to mitigate the pathogenic effects of *TARDBP* mutations. The study by Nishimura et al. found that siRNAs selected for targeting the TDP-43^M337V^ mutation can reduce the levels of cytoplasmic TDP-43 in ALS patient-derived iPSCs [99]. Azpurua et al. employed the RNAi approach by using shRNAs to screen 2700 genes for transcripts whose knockdown could mitigate motor neuron dysfunction caused by TDP-43 overexpression [100]. They targeted key factors in ALS pathology and reduced the toxic effects of *TARDBP* mutations.

Kuespert et al. developed an ASO targeting TGFβ receptor II (*TGFBR2*) mRNA using an LNA(locked nucleic acid)-gapmer design to inhibit TGFβ signaling. After a series of processes including in-silico-design, in vitro screening for activity and toxicity, and optimization, the lead compound, NVP-13 was identified to confer targeted degradation of mutant mRNA of *TGFBR2* while preserving the expression of the wild-type allele [101]. These contributions highlight ASOs' strategic role in gene therapy, providing a precise approach to correct genetic dysfunctions in ALS and other diseases.

Altered RNA metabolism and RNA toxicity play a critical role in ALS, particularly in the context of *TARDBP* mutations. It is important to understand the complex interactions between RNA dysregulation and neurodegeneration in ALS. Targeting RNA metabolism could provide new avenues for ALS treatment [102]. Studies by Klim et al. and Sun et al. have elucidated the intricate connections between TDP-43 dysfunction, RNA homeostasis, and neurodegeneration, highlighting the crucial role of TDP-43 in ALS pathology [103, 104]. TDP-43 dysregulation impacts RNA processing, including splicing and transport, leading to neuronal damage. In-depth research on the role of TDP-43 in RNA metabolism and neuronal integrity can open avenues for targeted interventions in neurological diseases associated with *TARDBP* [103].

Chen et al. identified a new *TARDBP* mutation in ALS patients consistent with FTD and parkinsonism. Their findings revealed a broader phenotypic spectrum of *TARDBP* mutations, including ALS, FTD, and Parkinson's disease. This discovery not only sheds light on the genetic basis of these diseases but also underscores the need for targeted genetic screening in ALS patients exhibiting FTD and Parkinsonian syndrome symptoms, driving the field towards more precise diagnostic and therapeutic strategies [105].

When studying gene therapy strategies for ALS in the context of *TARDBP* mutations, the role of liquid–liquid phase separation (LLPS) in ALS pathogenesis should not be overlooked. TDP-43 produced in *TARDBP* mutant cells undergoes an abnormal LLPS process, forming stable, irreversible, and long-lived droplet aggregates, leading to pathological processes such as the inhibition of nucleocytoplasmic transport, clearance of nuclear TDP-43, and cell death [106]. A previous study by Conicella et al. discovered that the α-helical structure of TDP-43 functions as a short but unique tunable module that can regulate the LLPS process through its biophysical properties [107]. A more recent study further elucidates the molecular mechanisms by which LLPS regulates functional TDP-43 [108]. Additionally, various substances, such as the fluorescent molecule 4,4′-dialkyl-1,1′-dinaphthyl-5,5′-disulfonic acid (bis-ANS) and Congo red, which bind to hydrophobic regions of proteins, have been shown to induce LLPS. These compounds hold significant potential in regulating the homeostasis of LLPS [109, 110]. These studies suggest that LLPS could serve as a focal point for enhancing the efficacy of *TARDBP*-targeted therapies for ALS in the future.

The selection of experimental models is also important for development of gene therapy. A study by Layalle et al. delved into the genetic and molecular mechanisms of ALS, focusing on the TDP-43 and its *Drosophila* ortholog, TBPH, which has been used in several animal studies of neurodegenerative diseases [110–113]. Their research revealed the toxic effects of gain-of-function mutations in TBPH and TDP-43, which are dose- and age-dependent and require RNA binding to manifest toxicity. They explored potential molecular mechanisms of TDP-43 toxicity, including splicing inhibition, mitochondrial dysfunction, transposon upregulation, and excitotoxicity. Additionally, they identified genetic modifiers of TDP-43 toxicity, highlighting the involvement of stress granules, cytoplasmic aggregation, unfolded protein response, inflammation, the mechanistic target of rapamycin kinase (mTOR) pathway, autophagosomes, chaperones, chromatin, hnRNP, and metabolic dysregulation. This research emphasizes the importance of studying ALS genes in *Drosophila* models to uncover new pathogenic mechanisms and potential therapeutic targets [112].

RING Finger Protein 220 (RNF220), an ubiquitin E3 ligase, regulates multiple signaling pathways, such as Shh/Gli, and is involved in early neural patterning, and development of the cerebellum and the locus coeruleus. Loss of RNF220 inhibits proliferation and promotes differentiation of neural stem cells. RNF220 interacts with TDP-43, leading to its polyubiquitination and degradation. In RNF220 haploinsufficient mice, this process is reduced, resulting in elevated TDP-43 levels in the spinal cord [114]. The RNF220 haploinsufficient mice exhibit motor neuron defects, muscle denervation, atrophy, and TDP-43 accumulation in spinal motor neurons. These symptoms mimic the clinical and pathological phenotypes observed in human ALS [114]. Therefore, RNF220 may act as a modifier of TDP-43 function and contribute to the progression of ALS-like phenotypes. RNF220 haploinsufficient mice could serve as a potential model for understanding ALS mechanisms and testing therapies [114].

### *FUS*/*TLS (Translocated in Liposarcoma)*

*FUS*-ALS mutations cause a widespread loss of expression and splicing functions. Mutated FUS directly alters intron-retention levels in RNA-binding proteins. Intron retention events have been identified within FUS itself, which are related to its autoregulation. *FUS* mutations disrupt this autoregulatory mechanism. Importantly, increased FUS intron retention has been observed in other genetic forms of ALS, including those caused by mutations in *TARDBP*, *VCP*, and *SOD1*, supporting the concept of interaction between multiple ALS genes within regulatory networks [115]. Mutations in *FUS* account for approximately 4% of familial ALS cases and less than 1% of sporadic ALS cases, leading to ALS and certain forms of FTD through mislocalization of FUS to the cytoplasm. This mislocalization results in both gain-of-function and loss-of-function pathogenic mechanisms, as demonstrated by studies from Murakami et al. and Sharma et al. [116, 117]. These mutations induce the cytoplasmic accumulation of mutant FUS and the formation of neurotoxic aggregates, leading to neuronal dysfunction, synaptic hyperexcitability, and motor neuron degeneration. The study by Vance et al. further highlights the complexity of FUS pathogenicity, linking cytoplasmic mislocalization with synaptic defects and altered neuronal activity [117]. Understanding these mechanisms is crucial for developing targeted gene therapy strategies to address FUS-related neurodegeneration in ALS.

ALS-related FUS abnormally contacts U1 snRNA at the Sm site with its zinc finger domain, and traps snRNP (small nuclear ribonucleoprotein) biogenesis intermediates in motor neurons. This abnormal interaction represents a toxic gain of function, linking ALS to spinal muscular atrophy [118]. In addition to U1 snRNA, FUS also interacts with other snRNAs, such as U4, U5, and U6, during spliceosome assembly. These interactions, mediated by the RNA-recognition motif domain of FUS, suggest that FUS plays a role in guiding spliceosome assembly through various snRNA interactions [118]. Mutations in the *FUS* gene, which is involved in DDR and DNA repair, lead to increased DNA damage in FUS-ALS, likely due to defects in these processes [54].

Currently, research on gene therapy targeting *FUS* is underway. Sanjuan-Ruiz et al. discovered that introducing the human *FUS* gene into *Fus*-mutant mice activates the autoregulation of mutant *Fus* to reduce mutant FUS protein. The mechanisms involve the retention of introns 6 and 7 in the endogenous mouse *Fus* mRNA and reduction of the expression of mutant mRNA, thereby lowering cytoplasmic FUS levels and subsequently reducing mortality and motor defects of the *Fus*-mutant mice [119]. Tejido et al. found that histone deacetylase inhibitors affect the cytoplasmic localization of FUS, promoting the acetylation of the FUS RNA-binding domain and altering its interaction with RNA in *FUS*-ALS models [120]. Additionally, Jacifusen (ION363), an ASO targeting *FUS* mutations, can prevent the production of FUS protein by targeting *FUS* mRNA. This drug has entered a Phase 3 trial to determine its safety and efficacy in a cohort of 77 patients with *FUS* mutations in the US and the Europe [121]. However, challenges remain in the translation of results from transgenic mouse experiments to clinical application, including the need for gene vectors with more stable expression.

### *Ataxin 2 (ATXN2)*

The *ATXN2* gene, particularly its intermediate-length CAG trinucleotide repeat expansions, plays a crucial role in the pathogenesis of ALS. Research has demonstrated a significant association between these expansions and an increased risk of ALS, with certain alleles showing a strong correlation with disease susceptibility [122, 123]. The prevalence and impact of these expansions vary among populations, as evidenced by studies on Korean ALS patients [123]. Borghero et al. further revealed that the length of these expansions influences the ALS phenotype, with longer repeat numbers linked with spinal onset and shorter survival time [124]. Wang et al. highlighted intermediate CAG repeat expansion in *ATXN2* as a unique genetic risk factor for ALS, distinct from monogenic mutations [125].

*ATXN2* exhibits synergistic pathogenic relationships with other genes. Co-expression of ATXN2 intermediate polyglutamine repeat sequences (30Q) and *C9orf72* deletion increases ATXN2 aggregation and neuronal toxicity. These results were confirmed in zebrafish embryo experiments, where partial knockdown of *C9orf72* and expansion of intermediate (but not normal) ATXN2 repeats lead to motor deficits and abnormal axon projections of spinal motor neurons [77]. In addition, TDP-43 localization to ATXN2-dependent stress granules is a common pathological endpoint in ALS. Administration of ASOs targeting *ATXN2* in TDP-43 transgenic mice increases the survival rate [126, 127]. These studies suggest that therapeutic strategies targeting ALS-related risk genes might have broad applicability across different mutation types, warranting extensive experimental validation.

In addition to the therapies mentioned above, there are currently clinical trials underway targeting *ATXN2*. BIIB105 is an ASO against *ATXN2* that degrades *ATXN2* mRNA and reduces ATXN2 protein levels. It is currently undergoing a Phase I clinical trial to evaluate its safety and tolerability [128].

### *Matrin 3 (MATR3)*

Dysfunction of RNA-binding proteins is a fundamental hallmark of ALS and related neuromuscular disorders. MATR3 is an RNA/DNA-binding protein that interacts with TDP-43. Johnson et al. identified mutations in *MATR3* among ALS family members using exon sequencing [129]. Pathological MATR3 changes have been observed in sporadic ALS, highlighting its pivotal role in the pathogenesis of neuromuscular disorders [130]. Glutamatergic stimulation reduces MATR3 function through two complementary mechanisms. Within minutes of NMDA-induced depolarization, CaM translocates to the nucleus and the binding of Ca^2+^/CaM to MATR3 attenuates its ability to bind RNA. Over extended periods of time, glutamatergic activity drives MATR3 degradation through NMDA receptor-, Ca^2+^-, and calpain-dependent mechanisms that drive MATR3 degradation. The pathogenic mutant MATR3 is resistant to calpain degradation, potentially contributing to the onset of ALS-related pathological changes [130].

Interestingly, Rao et al. found that overexpression of the endogenous calcium protease inhibitor calpastatin in an SOD1 mutant ALS mouse model can prevent proteolytic cleavage targeting proteins like MATR, improving motor function and survival [131]. Ramesh et al. discovered that the *Drosophila* homolog of human RNA-binding protein hnRNPM, known as rump, acts as a modifier of mutant MATR3 to inhibit its toxicity. Experiments in mammalian cells revealed that hnRNPM physically and functionally interacts with MATR3 at least partially through binding to shared RNA targets [132]. While these studies elucidate the pathogenic role of MATR3 in ALS, there has been limited exploration of direct therapeutic targeting of the *MATR3* gene. Nevertheless, targeting MATR3 at the protein level for therapeutic efficacy remains a promising avenue of investigation.

### Other potential targets

In recent years, with the deepening of research into ALS-related risk genes, more genes have been revealed, providing a theoretical basis for experimental studies in the future. Below we summarize promising ALS-related risk genes that could become new targets for gene therapy.

### *Ubiquilin 2 (UBQLN2)*

UBQLN2 belongs to the ubiquilin family and is involved in protein degradation pathways, including the ubiquitin–proteasome system and the autophagy-lysosome pathway. In ALS, mutations in *UBQLN2* have been shown to be associated with disease progression, primarily due to protein aggregation, impaired protein degradation, stress response, and neuroinflammation. Deng et al. discovered that mutations in the *UBQLN2* gene lead to dominant, X-linked ALS and ALS/dementia. Functional analysis revealed that mutations in *UBQLN2* result in impaired protein degradation, suggesting a common pathogenic mechanism that could be targeted for therapeutic intervention [133].

Mutations in *UBQLN2* contribute to ALS pathogenesis not only through their intrinsic expression mechanisms but also by inducing gene regulatory abnormalities. Black et al. demonstrated that UBQLN2 regulates the domesticated gag-pol retrotransposon "paternally expressed gene 10 (PEG10)" in human cells and tissues. In cells, the PEG10 gag-pol protein cleaves itself through a mechanism similar to that of retrotransposon self-processing, generating a released "nucleocapsid" fragment that uniquely localizes to the nucleus and alters the expression of genes associated with axonal remodeling. In the spinal cord tissues of ALS patients, PEG10 gag-pol levels are higher than that in healthy controls. These findings indicate that the retrovirus-like activity of PEG10 is a mechanism by which gene expression regulation leads to ALS, and that inhibiting PEG10 is a major function of UBQLN2 [134].

### *NEK1*

*NEK1* is a gene encoding a serine/threonine kinase involved in various cellular functions, including cilia formation, DDR, microtubule stability, neuronal morphology, and axon polarity, offering insights into the pathogenesis and genetic etiology of ALS [135]. As part of the NIMA kinase family, NEK1 plays roles in cell cycle progression, mitosis, ciliogenesis, mitochondrial membrane permeability, and DNA repair. Disruptions in these functions are linked to various neurological defects and diseases, affecting 2%–3% of ALS cases [135]. Mutations in NEK1 impair its role in the DDR, leading to increased DNA damage in *NEK1*-ALS [54]. Kenna et al. identified a significant association between *NEK1* loss-of-function variants and familial ALS risk, with the NEK1 p.Arg261His variant being a candidate risk factor [135].

Mann et al. found that NEK1 interacts with proteins related to cytoskeletal homeostasis, nucleocytoplasmic transport, and protein homeostasis. Key interacting factors include α-tubulin (TUBA1B) and importin-β1 (KPNB1), both phosphorylated by NEK1. Therefore, NEK1 plays a critical role in microtubule dynamics and nuclear import processes [136]. Reduced NEK1 levels in motor neurons cause disruption of microtubule homeostasis. NEK1 interaction with TUBA1B and its ability to phosphorylate TUBA1B indicate that NEK1 regulates microtubule stability. NEK1 loss-of-function results in reduced TUBA1B retention and impaired microtubule polymerization, affecting neurite outgrowth and other downstream processes [136]. NEK1 loss-of-function also impairs nucleocytoplasmic transport. Reduced NEK1 levels lead to decreased KPNB1 intensity in the nucleus and along the nuclear membrane, indicating impaired nuclear import. Experiments using a fluorescent tdTomato reporter gene further confirmed that NEK1 knockdown results in the translocation of the reporter signal from the nucleus to the cytoplasm [136].

In terms of treatment, *NEK1* has recently received attention as an ALS-related gene, and current therapies mainly focus on the protein level. Mann et al. demonstrated that stabilizing microtubules with drugs such as paclitaxel and laulimalide can improve NEK1-dependent microtubule homeostasis and nuclear import defects. This suggests a potential therapeutic approach to address the pathogenic effects of NEK1 loss-of-function in ALS [136].

### *Splicing factor proline and glutamine rich (SFPQ)*

The loss of SFPQ is a hallmark of ALS motor neuron degeneration. Huang et al. found that nuclear depletion and cytoplasmic mislocalization of SFPQ are associated with ALS neuropathology [137]. Gordon et al. reported that the loss of SFPQ results in premature termination of multiple transcripts due to the widespread activation of previously unannotated cryptic last exons (CLEs). These CLEs, which are repressed by SFPQ, preferentially occur in long introns of genes with neuronal functions and inhibit gene expression outputs and/or produce short peptides that interfere with normal gene functions. The researchers showed that the peptide encoded by the CLE-containing *epha4b* mRNA is responsible for the neurodevelopmental defects in SFPQ mutants. The CLE-suppressing activity of SFPQ is conserved in both mice and humans, and SFPQ-repressed CLEs are expressed in ALS iPSC-derived neurons. These findings significantly expand our understanding of SFPQ function and reveal a gene regulatory mechanism broadly relevant to human neuropathology [138].

Additionally, the normal nuclear interaction between SFPQ and FUS is crucial for neuronal homeostasis. Shinsuke et al. found that disruption of the FUS–SFPQ interaction leads to an increased ratio of 4-repeat tau to 3-repeat tau, which is a common phenotype in the ALS spectrum [139]. This further suggests that combined multi-targeting gene therapy for ALS might become a significant therapeutic strategy in the future.

### *Collapsin response mediator protein 4 (CRMP4)*

CRMP4 is a class of developmentally regulated phosphoproteins. Maimon et al. found that alterations in CRMP4 protein levels lead to motor neuron loss in ALS. They observed an increase in CRMP4 in the cell bodies of motor neurons affected by ALS but a decrease in the distal axons. This mislocalization of CRMP4 is promoted by an increased interaction with the retrograde motor protein dynein, facilitating the transport of CRMP4 from distal axons to the soma, which is associated with motor neuron loss. Blocking the CRMP4–dynein interaction reduces motor neuron loss in human-derived motor neurons and ALS model mice, suggesting a novel CRMP4-dependent retrograde death signal underlying motor neuron loss in ALS [140].

Although there are currently no clinical trials targeting *CRMP4* for ALS, early animal studies have suggested that this gene could be a promising target for ALS treatment. In a study by Duplan et al., AAV-mediated overexpression of *CRMP4a* resulted in the death of 30% of lumbar motor neurons and an 18% increase in denervation at the neuromuscular junctions of the gastrocnemius muscle in wild-type mice. Conversely, silencing *CRMP4a* protected *SOD1* mutant motor neurons from NO-induced death [141].

### *VCP*

VCP is a ubiquitously expressed AAA + ATPase involved in multiple processes such as protein degradation, DNA repair, apoptosis, and autophagy, and is directly associated with ALS [142]. Hung et al. demonstrated that familial *VCP* mutations lead to abnormal interactions between FUS and SFPQ (a splicing factor), ultimately resulting in spatial dissociation. These processes are associated with disrupted alternative splicing of *MAPT* (microtubule-associated protein tau) pre-mRNA and increased tau phosphorylation in human *VCP* mutant cortical neurons. In addition, increasing 4R tau via the ASO technology is sufficient to drive neurodegeneration in human cortical neurons. This suggests that ASOs targeting VCP might be a promising gene therapy approach for ALS [143].

The above studies highlight the interaction of VCP with other common ALS risk genes. Harley et al. and Ziff et al. found that ML240, which inhibits the D2 ATPase domain of VCP, reduced the nuclear-to-cytoplasmic mislocalization of TDP-43, FUS, SFPQ, and several other proteins and their mRNAs in spinal motor neurons derived from human-induced pluripotent stem cells (hiPSCs) carrying *VCP* mutations [144, 145]. These results underscore the importance of interactions among ALS risk genes in future gene therapy research, suggesting that gene therapies targeting overlapping pathways might yield promising results.

## Autophagy-related potential targets

Autophagy plays multiple roles in neurodegenerative diseases by clearing harmful proteins, regulating neuroinflammation, and protecting mitochondria [146]. Autophagy has diverse functions in the progression of ALS. It not only serves as a potential target for gene therapy, but also helps cells effectively manage newly synthesized proteins post-therapy, preventing their misfolding and aggregation, thereby enhancing the therapeutic outcomes. Here, we introduce several autophagy-related genes involved in ALS, in order to provide new directions for gene therapies targeting autophagy in ALS.

### TBK1

Autophagy is intricately regulated by various autophagy-related (ATGs) proteins. The TBK1 pathway is a downstream effector of autophagy initiator beclin-1 [147]. In addition, TBK1 colocalizes with the autophagy receptor optineurin and cellular aggregates in vivo in *SOD1* transgenic ALS mouse models [147]. Cirulli et al. found significant enrichment of TBK1 mutations in ALS patients, including loss-of-function mutations such as nonsense, splice site, frameshift, and deletions [148], leading to autophagic defects that promote progressive accumulation of protein aggregates and drive ALS progression [147].

The pathogenic impact of TBK1 loss-of-function mutations in SOD1 mice typically requires a second hit at the genetic level [149]. *TBK1* variant carriers often harbor additional mutations in other ALS-related genes, suggesting an oligogenic model of pathogenesis in sporadic ALS [150, 151]. Individuals carrying both *C9orf72* repeat expansions and *TBK1* mutations frequently exhibit earlier and more rapid onset of ALS [150–153]. The abnormal DRP Poly(GA), arising from *C9orf72* gene mutations, can sequester TBK1, leading to reduced TBK1 function, impaired endolysosomal maturation, and induction of TDP-43 aggregation. However, Shao et al. demonstrated that overexpression of TBK1 can reduce Poly(GA) aggregation, highlighting the potential of TBK1-targeted therapy for ALS [154].

Besides its role in autophagy, TBK1 acts as an endogenous inhibitor of receptor-interacting protein kinase 1 (RIPK1) that promotes age-dependent neurodegeneration and contributes to ALS onset. Xu et al. further highlighted another endogenous RIPK1 inhibitor, TAK1, involved in ALS.  Downregulation of TAK1 in *TBK1* mutant mice exacerbates various hallmark features of ALS such as neuroinflammation and TDP-43 aggregation. Targeting these genes may thus offer potential avenues for mitigating the progressive course of ALS [155].

#### *OPTN*

OPTN, as a selective autophagy receptor, identifies and binds ubiquitinated proteins and damaged organelles such as mitochondria, transporting them to autophagosomes for degradation [156]. OPTN deficiency contributes to progressive myelin dysregulation and axonal degeneration through engagement of RIPK1, RIPK3, and MLKL (mixed lineage kinase domain-like protein). In addition, inhibiting RIPK1 kinase activity could provide a strategy for axonal protection in ALS [157].

Maruyama et al. discovered various *OPTN* mutations in ALS patients. Nonsense and missense mutations of *OPTN* eliminate its inhibitory effect on nuclear factor kappa B (NF-κB) activation. They also found that NF-κB inhibitors could be used to treat ALS, and transgenic mice carrying various *OPTN* mutations can be utilized to develop new therapies for this disease. This study highlights potential indirect targets for gene therapy in ALS [158].

The interaction between *OPTN* and *TBK1* mutations is a complex and critical aspect of ALS pathology and a potential gene therapy target. TBK1 plays a role in autophagy and neuroinflammation. *TBK1* mutations impair autophagy, leading to protein aggregation in ALS [159, 160]. OPTN is commonly associated with autophagy dysfunction in neurodegenerative diseases, and its mutations in ALS can result in loss of function, leading to the activation of the NF-κB inflammatory signaling pathway [161, 162]. The functions of OPTN in selective autophagy are tightly regulated by TBK1. Li et al. have provided structural insights into *OPTN* mutations and their impact on OPTN recognition of ubiquitin and its regulation by TBK1 [163, 164]. These findings underscore the complex relationship between OPTN and TBK1 in ALS, emphasizing the importance of targeting these genes and pathways in the development of ALS gene therapies.

### *Sequestosome 1* (*SQSTM1*)

*SQSTM1* encodes the autophagy receptor p62. A whole-exome sequencing study has identified *TBK1* along with two autophagy genes, *OPTN* and *SQSTM1*, as susceptibility genes for ALS [148]. Protein misfolding and aggregation are features of ALS, suggesting defects in protein degradation mechanisms. Autophagic impairment leads to accumulation of p62, which negatively impacts DNA repair in ALS models [54].

In the progression of ALS, mutations of genes such as *FUS* lead to the aberrant formation of stress granules, a significant pathological feature of ALS. Chitiprolu et al. demonstrated that under normal conditions, C9orf72 binds to the autophagy receptor p62 and facilitates the autophagic clearance of aberrant stress granules. However, in ALS patients with *C9orf72* repeat expansions, symmetric arginine-dimethylated proteins accumulate, co-localized with p62, resulting in impaired autophagic function of both [165].

Impaired autophagic function leads to the failure of timely clearance of damaged mitochondria, which may initiate inflammatory cascades and activate injury signaling. A recent study revealed that NF-κB essential modulator (NEMO) is recruited to damaged mitochondria in a parkin-dependent manner. Upon recruitment, NEMO not only forms aggregates distinct from OPTN, but also co-localizes with p62/SQSTM1, facilitating the phosphorylation of IKKβ (inhibitor of kappa B kinase beta), a catalytic inhibitor of active IKK components, thereby initiating NF-κB signaling transduction and upregulating inflammatory cytokines. This process resembles neuroinflammation and promotes ALS progression. Given the co-localization of OPTN with NEMO, further investigation into their functional interactions is warranted. Whether this feature can be leveraged for targeted OPTN therapy remains to be validated [166].

### *ATG5 and ATG7*

ATG5 and ATG7 are critical proteins in the autophagy process. In mice deficient in ATG5, the formation and spread of pathological TDP-43 in the CNS significantly exacerbate ALS-like phenotypes along the corticospinal tract axons [167]. Mouse and *Drosophila* ALS models lacking TARDBP/TBPH demonstrate reduced ATG7 levels and accumulation of SQSTM1/p62 aggregates. Supplementation of ATG7 using the *Act5C*-*Gal4* driver in fruit fly models significantly suppresses the semi-lethal phenotype without affecting TBPH loss or expression. Moreover, overexpression of ATG7 markedly reduced SQSTM1 aggregates. These findings suggest that targeting ATG7 holds promise for treating ALS associated with other genetic mutations [168].

### *CHMP2B*

CHMP2B is subunit of ESCRT-III (Endosomal Sorting Complex Required for Transport-III), which is involved in the endolysosomal pathway and autophagy processes [169]. Early studies primarily associated *CHMP2B* gene mutations with FTD. Later, *CHMP2B* mutations were found in ALS and FTD patients [170]. *CHMP2B* mutations are likely pathogenic in ALS [171]. A recent study revealed that partial knockdown of non-muscle MYH10 (Myosin Heavy Chain 10)/myosin IIB/zip rescued neurodegeneration in *Drosophila* and human iPSC-derived cortical neurons expressing FTD-related mutant CHMP2B. This provides a potential strategy for indirectly targeting autophagy-related genes in ALS therapy [172].

### Immune-related potential targets

**Fig. 4:**
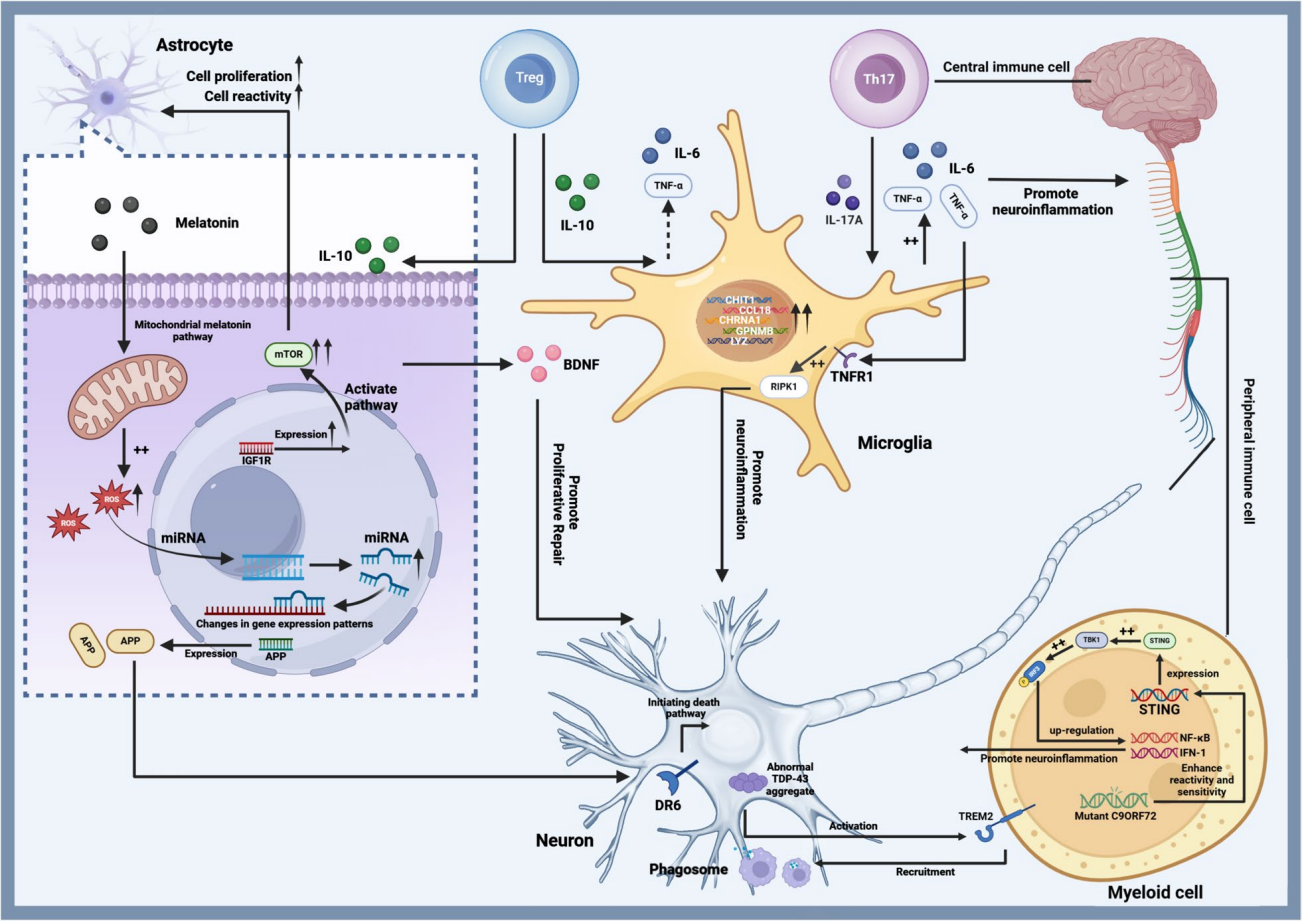
Pathogenesis and therapeutic potential of immune cells in ALS. Th17 cells produce IL-17A, activating microglia and astrocytes, leading to inflammatory responses that damage neurons in ALS. Treg cells secrete IL-10, slowing ALS progression by inhibiting neuroinflammation, regulating glial activity, promoting neuroprotection, and modulating immune responses. In astrocytes, the mitochondrial melatonin pathway inhibits pro-inflammatory factors, limiting inflammatory responses. Its reduction in ALS patients increases reactive oxygen species. The IGF1R-mTOR pathway is upregulated, inhibiting autophagy and enhancing astrocyte reactivity. In a pathogenic model, soluble APP fragments released by astrocytes activate death receptor 6 on motor neurons, triggering death signals through an NF-κB1-dependent pathway. In microglia, activation-related genes (CHIT1, CCL18, CHRNA1, GPNMB, LYZ) are upregulated in ALS. The RIPK1 pathway, activated by TNFα signaling, polarizes microglia into an inflammatory state, contributing to neurodegeneration. Myeloid cells in ALS show TREM2 receptor-mediated formation of phagocytic TDP-43 aggregates. Inhibition of STING reduces TDP-43-induced neurodegeneration by downregulating inflammatory genes through inhibition of TBK1 and IRF3 phosphorylation. C9orf72 knockout enhances myeloid cell sensitivity to STING, inducing an overactive type I IFN response. These cellular interactions and pathways contribute to neuroinflammation, neuronal damage, and disease progression in ALS. Understanding these mechanisms provides potential therapeutic targets aimed at slowing disease progression and protecting motor neurons. Figure created with BioRender.com

The immune system plays potential pathophysiological roles in ALS. Marchi et al. studied local and systemic factors (blood, cerebrospinal fluid, microbiota) influencing ALS progression in animals and humans. They also explored positron emission tomography for detecting neuroinflammation and reviewed immune-targeting clinical trials. These findings provide key insights into ALS immunology and, with further human validation, may inform new therapeutic targets and personalized treatments [173]. Here, we will summarize pivotal immune cells in ALS as well as promising gene therapy approaches in immunology research. The pathogenic mechanisms and therapeutic potential of immune cells in ALS are presented in Fig. 4.

### T cells

T cells, particularly helper T (Th) cells and regulatory T cells (Tregs), play a crucial immunological role in ALS [174, 175]. Studies have shown increased proportions of Th1 cells (secreting IFN-γ) and Th17 cells (secreting IL-17) in ALS patients, which promote inflammatory responses and neuronal damage. Specifically, Th17 cells, through production of IL-17A acting on various resident cells in the CNS, have been implicated in the pathogenesis of several neurodegenerative diseases [176]. IL-17A-targeting drugs have been appearing as potential immunomodulatory therapies for neurodegenerative diseases, including ALS. In ALS, these therapies primarily aim to neutralize IL-17A or target its receptor to reverse its toxic effects. Despite ongoing debates regarding the specific mechanisms of Th17/IL-17A in such diseases, uncovering the molecular pathways of Th17/IL-17A in neurodegenerative diseases can identify appropriate targets for regulating these cellular processes [177]. However, it is noteworthy that current research has not yet elevated IL-17A targeting to the level of gene therapy.

In addition to Th cells, the signaling pathways of cytokines related to Tregs also significantly influence the progression of ALS. The primary cytokines involved in ALS are TGF-β and IL-10. Henkel et al. reported that both Treg counts and TGF-β expression levels are reduced in rapidly progressing ALS patients. However, an earlier study by Galbiati et al. found that in mice overexpressing human mutant SOD1, the expression of TGF-β1 is already up-regulated at the presymptomatic stage, and treatment with the anabolic/androgenic steroid nandrolone further exacerbated the increase of TGF-β1 expression levels [178, 179]. Importantly, two later studies point out that excess TGF-β1 derived from astrocytes can accelerate ALS disease progression by disrupting autophagy and other neuroprotective mechanisms [180, 181]. Therefore, TGF-β may have different effects on ALS progression depending on its cellular source and context, and its precise mechanisms require further investigation.

IL-10 suppresses the pro-inflammatory activity of microglia by downregulating the expression of inflammatory mediators such as TNF-α and IL-6. Furthermore, IL-10 enhances the neuroprotective capacity of astrocytes, stimulating the secretion of neurotrophic factors like BDNF, which plays a crucial role in neuronal survival and function [181–184]. In a study by Ayers et al., rAAV vectors were used to induce overexpression of IL-10 in Tregs with a predominantly anti-inflammatory phenotype in SOD1^G93A^ mice. IL-10 slowed the progression of ALS without significantly affecting disease onset [185]. Gravel et al. further found that prolonged infusion of IL-10 receptor blocking antibodies could trigger the onset of ALS symptoms in mice. In contrast, gene therapy approaches that induce overexpression of IL-10 in microglia significantly delayed disease onset and improved survival rates in ALS mouse models [186]. Clinical trials have demonstrated the safety and tolerability of IL-10, thereby enhancing its potential as a therapy for ALS and other motor neuron diseases. Unlike therapies targeting IL-17A as mentioned above, this research targets IL-10 therapy for ALS at the gene expression level. Therefore, further research is warranted to explore how to target T cell IL-17A-related genes and suppress their expression through viruses or other vectors in the field of ALS immunogene therapy.

### Astrocytes

Astrocytes are implicated in various aspects of ALS pathogenesis, including neuroinflammation and glutamate toxicity [187]. Anderson’s research demonstrates that the melatonin pathway in astrocytes can inhibit the activation of pro-inflammatory transcription factors (such as NF-κB and Yin Yang 1) induced by Toll-like receptor agonists. This inhibition helps restrict inflammation by promoting the synchronized release of melatonin. In ALS patients, there is a degree of dysfunction in the melatonin pathway within astrocytes. This decline results in persistent activation of astrocytes often accompanied by enhanced microglial reactivity, a critical driver of motor neuron susceptibility in ALS. It is noteworthy that the mitochondrial melatonin pathway in astrocytes serves as a core aspect of cellular function, and its inhibition can increase reactive oxygen species (ROS), leading to microRNA changes that alter BDNF and other gene induction patterns [49]. Therefore, understanding how inhibition of the mitochondrial melatonin pathway in astrocytes affects these gene induction patterns in ALS patients and leveraging these insights for ALS gene therapy represent a promising direction for future research.

The mTOR pathway plays a role in the pathogenesis of ALS through astrocytic gliosis. In human-derived astrocytes carrying *SOD1* mutation, upstream positive regulators of the mTOR pathway, such as insulin-like growth factor 1 receptor (IGF1R), are significantly elevated at the transcription level. This elevation improves mTOR pathway activation, leading to increased autophagy inhibition, enhanced cell proliferation, and heightened astrocytic reactive responses. Inhibition of the IGF1R-mTOR pathway leads to decreased proliferation and reactivity of mutant SOD1 astrocytes, alleviating their toxicity towards motor neurons. These findings suggest that targeting the expression of the IGF1R-mTOR pathway in astrocytes may represent a viable gene therapy strategy for *SOD1* ALS and other potential neurodegenerative diseases of the nervous system [188].

Mishra et al. have systematically elucidated and evaluated the Regulatory Cell Interaction Network method to identify ligand-mediated interactions between different cellular compartments. They predicted and experimentally confirmed that the interaction between amyloid precursor protein (APP) in astrocytes and death receptor 6 (DR6) in motor neurons triggers astrocyte-mediated death signaling in motor neurons, highlighting their interaction as a ligand-receptor pair. RNAi-mediated knockdown of DR6 in motor neurons alleviated neurodegeneration in mouse models. This study proposed a pathogenic model for ALS neurodegeneration, where astrocyte-specific release of soluble APP fragments activates DR6 on motor neuron surfaces, triggering death signals through an NF-κB1-dependent pathway leading to spinal motor neuron death [189]. Additionally, the study suggested that lowering DR6 levels in motor neurons of transgenic mutSOD1 mice attenuates ALS-like phenotypes. Therefore, gene therapies in astrocytes, such as those targeting APP-related genes, could be considered for ALS treatment in the future.

### Microglia

Microglia in ALS mediate inflammatory responses and neuroprotection, potentially exacerbating neuronal damage or promoting neuronal survival under certain conditions [190]. A study using quantitative trait loci (QTL) mapping revealed increased gene expression in microglia and astrocytes, as well as decreased gene expression in oligodendrocytes in ALS patient samples. Genes such as *CHIT1*, *CCL18*, *CHRNA1*, *GPNMB*, and *LYZ*, which encode proteins secreted by activated microglia, were significantly upregulated in the ALS group. This study was the first to identify *CCL18* and *LYZ* as risk genes for ALS. In addition, molecular QTL map identified several potential ALS risk loci that may function through gene expression or splicing in the spinal cord. Some putative cell types were assigned for *FNBP1*, *ACSL5*, *SH3RF1*, and *NFASC* [191]. These findings provide numerous reference points for future development of gene therapies for ALS, highlighting the differential gene expression among various central immune cells in ALS and laying the groundwork for precise immune cell gene therapies.

Mifflin et al. identified a microglial subtype in an ALS mouse model, termed RIPK1-regulated inflammatory microglia (RRIM). These microglia exhibit significant upregulation of classic pro-inflammatory pathways, including increased RNA and protein levels of *Tnf* and *Il1β*. RRIM are highly regulated by TNFα signaling. In the SOD1^G93A^ mouse model of ALS, treatment with the RIPK1 inhibitor NEC-1 significantly reduced RRIM prevalence, indicating a reduction in pro-inflammatory microglia [192]. In the context of ALS pathogenesis, TNFα signaling polarizes microglia to the RRIM state, thereby enhancing the pro-inflammatory environment and contributing to neurodegeneration. Therefore, RIPK1 inhibitors might offer a broad approach to mitigating neuroglia-mediated inflammation in chronic neurodegenerative diseases like ALS. RRIM could also serve as a potential biomarker of RIPK1 activation in ongoing clinical trials of RIPK1 inhibitors for ALS [192]. While current RIPK1 inhibitors like NEC-1 primarily function at the protein level, future efforts to enhance inhibition efficiency and stability may involve developing *RIPK1*-related gene therapies.

### Myeloid cells

When studying immunotherapy for ALS, it is essential to consider myeloid cells. The stimulator of interferon genes (STING) pathway can induce neuroinflammation and interact with various ALS risk genes to exert pathogenic effects [193]. The STING pathway plays a crucial role in the pro-inflammatory actions of both central and peripheral immune cells. Among peripheral immune cells, myeloid cells have a significant relationship with the STING pathway. The phagocytosis of aggregates of TDP-43, a pathological hallmark of ALS, is triggered through the triggering receptor expressed on myeloid cells 2 (TREM2) receptor on myeloid cells [194]. Additionally, genetic deletion and pharmacological inhibition of STING can mitigate TDP-43-induced neurodegeneration by downregulating the expression of inflammatory NF-kB and type I IFN [195]. McCauley et al. found that C9orf72 is required for STING degradation through the autolysosomal pathway. Complete knockout of C9orf72 in mice increases the sensitivity of myeloid cells to activators of STING and induces an overactive type I interferon response. Blocking STING suppresses this type I interferon response, further confirming the interaction between STING and ALS pathogenic genes in myeloid cells [196]. These findings collectively underscore the potential of targeting STING-related genes in peripheral myeloid cells as a therapeutic strategy for ALS.

Selection of an appropriate research model is crucial for understanding the complex genetic variations within human immune cells in ALS. hiPSCs are a valuable tool for studying astrocyte-related ALS pathology. Many protocols have been established for generating astrocytes from patient-derived hiPSCs and neural progenitor cells [197]. However, this model has certain limitations. For example, various immune cells derived from hiPSCs exhibit fetal transcription profiles, complicating the study of age-related neurodegenerative diseases using hiPSC models [198]. Further research is needed to overcome these issues.

## Lipid-related gene targets

**Fig. 5:**
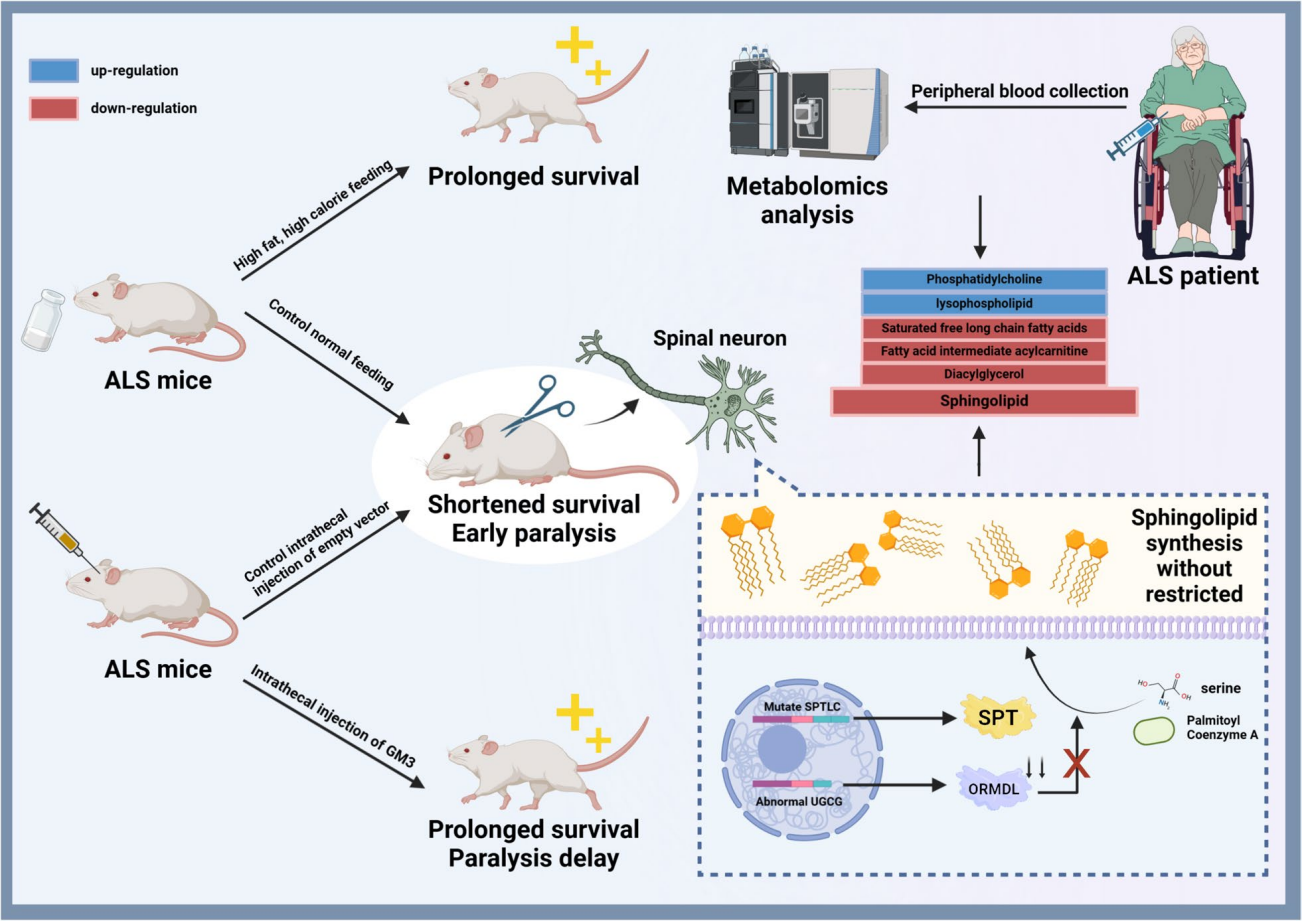
Pathogenesis and therapeutic potential of lipids in ALS. In mouse experiments, hyperlipidemia induced by high-energy diets significantly increases the survival rate of ALS G93A mice. Intraventricular infusion of GM3 ganglioside delays the onset of paralysis in SOD1^G93A^ mice. Human metabolomics studies have identified shared lipid pathways in independent ALS cohorts, including significant decreases in certain phosphatidylcholines and lyso-phospholipids, elevated levels of saturated long-chain free fatty acids, β-oxidation-related fatty acid intermediates such as acylcarnitines, and diacylglycerols. Elevated levels of sphingolipids, including sphingomyelins, ceramides, and cholesterol, are observed in the spinal cords of ALS patients and SOD1^G93A^ mice. Specific variants in the *SPTLC1* gene lead to loss of ORMDL-mediated inhibitory control over the SPT complex, resulting in unrestricted synthesis of sphingoid bases. Figure created with BioRender.com

Hyperlipidemia is a protective factor for ALS. Dupuis et al. found that hyperlipidemia induced by a high-energy diet significantly increased the survival rate of G93A SOD1 mice [199]. Similarly, Dorst et al. conducted a prospective interventional study showing that a high-fat, high-calorie diet can alleviate disease progression in ALS patients [200]. The role of lipids in the pathogenic mechanisms of ALS and their potential as targets of gene therapy is presented in Fig. 5.

To further explore the roles of lipids, Goutman et al. performed a metabolomics study and identified shared lipid pathways in an independent ALS cohort [201]. The study revealed a significant downregulation of complex lipids in ALS, including certain phosphatidylcholines and lysophospholipids. This alteration resulted in widespread fatty acid uptake, mitochondrial dysfunction, and abnormalities in multiple lipid signaling pathways. Moreover, elevated levels of saturated free long-chain fatty acids, β-oxidation-related fatty acid intermediates such as acylcarnitines, and diacylglycerol were observed in ALS patients. Metabolic pathway analysis further characterized ALS as a "hypermetabolic" state, primarily manifesting as increased resting energy expenditure, which may be associated with high glucose uptake and low fat oxidation [201]. Given these lipid metabolic changes, modulating lipid metabolic flux at the genetic level is a promising direction to alleviate ALS symptoms in future research.

It is particularly important to consider the role of sphingolipids in ALS. Sphingolipids constitute nearly 20% of the lipid content of the nervous system and participate in various physiological and developmental processes. Unlike metabolic lipids, sphingolipids (such as sphingomyelin, ceramide, and cholesterol) are abnormally elevated in the spinal cords of both ALS patients and SOD1^G93A^ mice [202]. Interestingly, subsequent studies found that inhibiting glucosylceramide synthesis significantly accelerated disease progression in SOD1^G93A^ mice, whereas intracerebroventricular infusion of GM3 ganglioside significantly delayed the onset of paralysis in these mice [203]. This indicates the complex role of sphingolipid metabolism in ALS pathogenesis, warranting further in-depth research. Moreover, some studies have begun to focus on the genetic aspects of sphingolipid metabolism. Henriques et al. focused on the UDP-glucose ceramide glucosyltransferase (*UGCG*) gene, which encodes the enzyme responsible for glucosylceramide synthesis. They found that applying a UGCG inhibitor significantly delayed functional recovery after sciatic nerve injury in an ALS mouse model [204]. Serine palmitoyltransferase (SPT) is the initial and rate-limiting enzyme in sphingolipid biosynthesis, encoded by the serine palmitoyltransferase long chain base subunit (*SPTLC*) genes. This synthesis process is tightly regulated by proteins such as the Ormds (ORMDL) family member to ensure adequate sphingolipid levels while preventing cytotoxicity from excessive accumulation. Mohassel et al. identified specific variants in the *SPTLC1* gene that cause the loss of ORMDL-mediated inhibitory control over the SPT complex, leading to unrestricted synthesis of sphingolipid bases and resulting in a monogenic form of ALS manifesting in childhood [205]. Johnson et al. expanded the phenotypic spectrum associated with *SPTLC1* and suggested screening for *SPTLC1* mutations in juvenile ALS patients [206]. Thus, targeting *SPTLC* for the treatment of childhood ALS could be a promising strategy.

## Cytoskeleton-related gene targets

The cytoskeleton, comprising microtubules, actin filaments, and intermediate filaments, plays a pivotal role in a myriad of cellular functions. In ALS models, significant damage to the cytoskeleton has been observed. ALS-associated gene mutations, such as *SOD1* and *C9orf72* mutations, can markedly affect cytoskeletal function. This raises interest in targeting the functional roles of cytoskeleton-related genes in ALS [207].

Currently, only eight key cytoskeleton-related genes are directly implicated in ALS, including α-tubulin (*TUBA4A*), *SPAST* (spastin), *KIF5A* (kinesin family member 5A), dynactin-1 (*DCTN1*), *NF* (neurofilament), *PRPH* (peripherin), alsin (*ALS2*), and *PFN1* (profilin). Their proteins play crucial roles in intracellular transport and signaling. Mutations in these genes can impair motor neuron function through mechanisms such as disrupting intracellular transport, forming abnormal protein aggregates, and causing protein misfolding, ultimately leading to ALS [207]. The peptide drug GM604 (also known as GM6 or Alirinetide) acts both as a neurotrophic factor and by modulating various genes associated with microtubule stability and ALS, such as *TUBA4A* and *NEFL* (neurofilament light chain), showing great potential in ALS gene therapy [208]. GM604 has also demonstrated safety and promising effects in a small phase IIA clinical study [209]. While most current studies primarily focus on the basic functions of cytoskeleton-related genes, the findings underscore the therapeutic promise of such approaches.

## Conclusion

ALS is a heterogeneous neurodegenerative disorder, influenced by age, sex, genetics, and ethnicity, leading to diverse clinical phenotypes. Studies have shown that older females are more likely to develop bulbar-onset ALS, while males tend to present with leg- or respiratory-onset forms [210]. Additionally, variations in ALS across ethnic groups, such as differences in onset age and bulbar-onset frequency between German and Chinese cohorts, further emphasize the complex nature of the disease [211]. Mutations in genes including *ALS2*, *DCTN1*, *MATR3*, and *OPTN* contribute to distinct clinical manifestations, emphasizing the need for personalized treatments [6, 212]. The pathogenic mechanisms of ALS-related genes are summarized in Table 3. However, current understanding of the genetic-environmental interactions in ALS remains incomplete, making it challenging to develop universal gene therapies.

**Table 3:** Pathogenic mechanisms of ALS-related genes

Gene types	ALS-related risk genes	Pathogenic mechanisms in ALS
Classic ALS risk genes currently undergoing gene therapy clinical trials	*SOD1*	Aggregation of misfolded SOD1 protein [51] Induction of DNA oxidative damage [54] Activation of motor neuron ferroptosis [59]
	*C9orf72*	Functional deficits leading to axonal transport defects and increased apoptosis, as well as decreased levels of HSP70 and HSP40 [69] Production of DPRs from transcription of HRE or repeat-associated non-AUG translation, causing toxic gain of function [69] Functional deficits leading to impairment of the autophagy-lysosome pathway, affecting clearance of DPR proteins and leading to their toxic accumulation [75] Production of DPRs can impair nucleocytoplasmic transport, including that of TDP-43, by interfering with the functions of RanGAP and NIR [70–73]
	*TARDBP*	Increased DNA damage due to impaired DDR signaling or DNA repair [54] Risk variants in *UNC13A* show significant levels of selective splicing and increased inclusion of cryptic exons when normal TDP-43 is depleted, further leading to nonsense decay and loss of UNC13A protein [89, 90] TDP-43 binding with PAR exhibit abnormal localization and aggregation, leading to neurodegenerative disease pathology [97] Lack of TDP-43 results in reduced ATG7 and accumulation of SQSTM1/p62 bodies, causing autophagic dysfunction [168] The loss of TDP-43 results in reduced STMN2 expression, which in turn impairs the axonal regeneration capacity of motor neurons [91–95]
	*FUS/TLS*	FUS mutations lead to widespread loss of its auto-regulatory functions in expression, splicing, and intron-associated regulation, directly altering intron retention levels of RNA-binding proteins [116, 117] Mutant FUS causes cytoplasmic accumulation and neurotoxic aggregates, resulting in neuronal dysfunction, synaptic hyperexcitability, and motor neuron degeneration [117] Mutant FUS aberrantly interacts with U1 snRNA at Sm sites within its zinc finger domains, disrupting the biogenesis of snRNPs in motor neurons, and leading to DDR and DNA repair impairments [118, 54]
	*ATXN2*	Intermediate-length CAG trinucleotide repeat expansions are associated with ALS onset and shorter survival times [121–124] Co-expression of ATXN2 with an intermediate polyglutamine repeat sequence (30Q) enhances aggregation and neuronal toxicity in conjunction with C9orf72 deletion [77] TDP-43 localization to ATXN2-dependent stress granules represents a common pathological endpoint in ALS [121, 122]
	*MATR3*	Mutations in MATR3, a RNA/DNA binding protein, affect its interaction with TDP-43 after expression, leading to various functional impairments in ALS [129, 130] Aberrant degradation of MATR3 protein diminishes its ability to bind RNA substrates, inducing pathological changes associated with ALS [130]
Classic ALS risk genes currently not undergoing gene therapy clinical trials	*UBQLN2*	Mutations in* UBQLN2* lead to defects in the ubiquilin-2 protein encoded by it, affecting protein degradation pathways, abnormal protein aggregation, and neurodegenerative changes [133] Inhibition of domesticated gag-pol retrotransposon PEG10 leads to aberrantly elevated PEG10 gag-pol expression after mutations, altering the expression of genes related to axon remodeling [134]
	*NEK1*	Mutations in *NEK1* result in functional deficits in cell cycle progression, mitosis, ciliogenesis, mitochondrial membrane permeability, DDR signaling, and DNA repair in the nervous system [135] NEK1 interacts with proteins related to cytoskeletal homeostasis, nucleocytoplasmic transport, and protein stability. Mutations disrupt the stability of cellular structures such as the cytoskeleton and microtubules, leading to impaired nuclear import [136]
	*SFPQ*	Deletion of SFPQ leads to widespread activation of CLEs, causing premature termination of multiple transcripts [138] Disruption of FUS-SFPQ interaction in the nucleus leads to an increased 4R-tau/3R-tau ratio, disrupting neuronal homeostasis [139]
	*CRMP4*	CRMP4 plays a role in regulating neuronal development. It increases in the cell bodies of motor neurons affected by ALS but decreases in distal axons, exacerbating motor neuron loss due to mislocalization [140]
	*VCP*	Mutations in *VCP* lead to abnormal interactions between FUS and SFPQ, ultimately causing spatial structural abnormalities in neurons [143] VCP is extensively involved in protein degradation, DNA repair, cell apoptosis, autophagy, and other processes. Mutations in VCP result in multiple neurologic functional deficits [142] *VCP* mutations often cause mislocalization of TDP-43, FUS, SFPQ, and a range of other proteins and their mRNAs from the nucleus to the cytoplasm [144, 145]
Autophagy-related genes	*TBK1*	The TBK1 pathway, downstream of autophagy initiator Beclin-1, when mutated, leads to autophagy defects causing progressive accumulation of protein aggregates, driving ALS progression [147, 148] Mutations in *TBK1* synergistically interact with *C9orf72* repeat expansions. Poly(GA) inclusions, abnormal dipeptide repeat proteins from C9orf72 mutations, can sequester TBK1, reducing its function, disrupting endosome maturation, and inducing TDP-43 aggregation [150–154] Mutations in *TBK1* result in impaired endogenous inhibition of RIPK1, promoting ALS onset [155]
	*OPTN*	Loss of OPTN contributes to necrotic apoptosis mechanisms in the central nervous system, ultimately leading to progressive myelin dysregulation and axonal degeneration [157] Nonsense and missense mutations in *OPTN* eliminate its inhibitory effect on NF-kB activation, promoting ALS progression [158] Mutations in *OPTN* impair its ability to recognize ubiquitin and mediate the degradation of abnormal organelles through autophagy in the nervous system [163, 164]
	*SQSTM1*	Autophagic dysfunction in the nervous system leads to accumulation of the autophagy receptor p62 encoded by *SQSTM1*, negatively affecting DNA repair in ALS models [54, 148] Mutant *SQSTM1* encodes abnormal p62, which is co-localized with mutant repeat expansion C9orf72, disrupts autophagic function, accelerating ALS progression [165] Autophagic dysfunction caused by mutant *SQSTM1* encoding abnormal p62 triggers NEMO-mediated inflammatory cascade reactions, exacerbating neuroinflammation [166]
	*ATG5, ATG7*	Defects in ATG5 lead to pathological formation and exacerbated spreading of TDP-43 in the central nervous system [167] Overexpression of *ATG7* significantly reduces accumulation of SQSTM1 bodies, whereas mutations and deficiencies exacerbate inclusion body formation [168]
	*CHMP2B*	Mutations in *CHMP2B* impair the endolysosomal pathway and autophagic processes, thereby promoting the progression of ALS [168–171]
Immune-related genes	*IL-17A*	Overexpression of aberrantly regulated genes can promote inflammatory responses and damage neurons [176]
	*TGF-β*	In different contexts, it may be possible to accelerate the progression of ALS disease by disrupting autophagy and other mechanisms, and may also have a role in slowing the progression of ALS [177–181]
	*IL-10*	Inhibition of multiple neuroinflammatory mediators such as TNF-αand IL-6 [181–184] Inhibition of the activation of microglia [181–184] Promoting the secretion of neuroprotective factors such as BDNF by astrocytes contributes to the survival and functional maintenance of neurons [181–184]
	*IGF1R*	Upregulation of the MTOR pathway leads to activation, resulting in inhibition of macroautophagy/autophagy, increased cell proliferation, and enhanced reactive astrocytosis [188]
	*DR6*	The protein expressed by *DR6*, acting as a receptor for APP, triggers death signals upon activation by astrocyte-specific release of soluble APP fragments. This activation occurs on motor neuron surfaces, leading to spinal motor neuron death through the NF-κB1-dependent pathway [189]
	*CHIT1*	Overexpression of *CHIT1* may reflect an inflammatory response to neurodegeneration, playing a significant role in the pathological progression of ALS [191]
	*CCL18*	Increased expression of *CCL18* may promote neuroinflammation and exacerbate neurodegenerative disorders [191]
	*CHRNA1*	Dysfunction of the α1 subunit of the nicotinic acetylcholine receptor (encoded by CHRNA1), which is involved in neurotransmission, has been implicated in the pathogenesis of ALS, potentially contributing to muscle weakness and atrophy [191]
	*GPNMB*	Regulates cell adhesion, migration, immune responses, and neuroinflammation. Aberrant expression can lead to dysregulation of inflammation [191]
	*LYZ*	Upregulation of *LYZ* may participate in neuroinflammation, promoting ALS [191]
	*TNF*	TNFα polarizes microglial cells to the RRIM state, thereby increasing pro-inflammatory environments and leading to neurodegenerative changes [192]
	*STING*	Interacts with various ALS risk genes, exerting pro-inflammatory effects in both central and peripheral immune cells [192–196]
Lipid-related genes	*UGCG*	Encodes glucosylceramide synthase. Inhibition of this gene can lead to prolonged recovery of function after nerve damage [204]
	*SPTLC*	The gene encodes SPTLC, which catalyzes the rate-limiting step in sphingolipid biosynthesis, converting serine and palmitoyl-CoA into sphinganine. Variations in this gene can disrupt the regulatory control of the SPT complex by ORMDL proteins, leading to uncontrolled sphinganine production, which contributes to the development of ALS [205]
Cellular cytoskeleton-related genes	*TUBA4A*	Regulates microtubule stability. Mutations can lead to an imbalance in microtubule homeostasis and axonal transport disruptions [207]
	*SPAST*	*SPAST* mutations may lead to abnormal microtubule severing function, thereby causing axonal transport defects and neurodegenerative disorders [207]
	*KIF5A*	Mutations in *KIF5A* may affect the normal function of motor proteins, leading to axonal transport disruptions and neurodegenerative disorders [207]
	*DCTN1*	Mutations in *DCTN1* may lead to dysfunction of the dynactin complex, affecting axonal transport and organelle localization [207]
	*NF*	Encodes neurofilaments. Mutations or abnormal expression may lead to instability in neurofilament structure, affecting axonal transport and neuronal survival [207]
	*PRPH*	Mutations in *PRPH* may lead to abnormal cell cytoskeleton structure and axonal transport impairments, thereby causing neuronal dysfunction [207]
	*ALS2*	Mutations in *ALS2* may affect the normal function of endosomes and axonal transport, leading to neurodegenerative disorders [207]
	*PFN1*	Mutations in *PFN1* may affect actin dynamics and cell cytoskeleton structure, leading to axonal transport disruptions and loss of neuronal function [207]

Early diagnosis is crucial to improve outcomes. Although current diagnostic criteria, such as El Escorial and Awaji, offer high specificity, they often result in delayed diagnoses. Modified criteria, like the Gold Coast standards, have better sensitivity but still struggle to detect slowly progressing cases [213, 214]. Despite advances in omics technologies that have identified several promising biomarkers (such as TREM2 and neurofilaments), their clinical value requires further validation [215, 216].

In ALS gene therapy, animal models remain essential for preclinical testing, but translating findings to humans is difficult due to anatomical differences, particularly in the motor system [217, 218]. Larger animal models, such as rhesus monkeys, offer more relevance to human disease than rodents, though they are more costly and less efficient [218–222]. For clinical trials, stratification based on phenotypic differences, such as disease progression and site of onset, is key to improving outcomes. Additionally, the use of biomarkers like neurofilament levels for patient stratification could help optimize trial results [217, 223, 224].

Currently, ALS gene therapies primarily target functional outcomes, including survival and muscle strength. However, challenges remain in determining the appropriate dosage for different muscle regions [225, 226]. Combining gene therapies with existing treatments, such as riluzole, which reduces glutamate toxicity, may enhance the therapeutic efficacy [208, 227, 228]. Nonetheless, the path to successful combination therapies and effective gene editing requires more refined models and better biomarkers to track progress and inform clinical decisions.

## Data Availability

Not applicable.

## Abbreviations

ALS: Amyotrophic lateral sclerosis
C9orf72: Chromosome 9 Open Reading Frame 72
SOD1: Superoxide dismutase 1
TARDBP: TAR DNA-binding protein
FUS: Fused in sarcoma
VCP: Valosin containing protein
TBK1: TANK-binding kinase 1
NEK1: NIMA related kinase 1
ASO: Antisense oligonucleotide
RNAi: RNA interference
siRNA: Small interfering RNA
RISC: RNA-induced silencing complex
GDNF: Glial cell line-derived neurotrophic factor
p-Akt: Phosphorylated Akt
IGF-1: Insulin-like growth factor 1
NDNF: Neuron-derived neurotrophic factor
iPSC: Induced pluripotent stem cell
shRNA: Short hairpin RNA
BBB: Blood–brain barrier
CNS: Central nervous system
P-gp: P-glycoprotein
LNPs: Lipid nanoparticles
TTC: Tetanus toxin heavy chain
MSCs: Mesenchymal stromal cells
BDNF: Brain-derived neurotrophic factor
NRF2: Nuclear factor erythroid 2-related factor 2
NfL: Neurofilament light chain
CSF: Cerebrospinal fluid
HRE: Hexanucleotide repeat expansion
FTD: Frontotemporal dementia
HSPs: Heat shock proteins
DPRs: Dipeptide repeat proteins
NIRs: Nuclear import receptors
RAB8: Ras-related protein Rab-8A
OPTN: Optineurin
SQSTM1: Sequestosome 1
HNRNPK: Heterogeneous nuclear ribonucleoprotein K
DDR: DNA damage response
UNC13A: Unc-13 Homolog A
STMN2: Stathmin-2
TGFBR2: TGFβ receptor II
LLPS: Liquid–liquid phase separation
RNF220: RING Finger Protein 220
MATR3: Matrin 3
UBQLN2: Ubiquilin 2
PEG10: Paternally expressed gene 10
SFPQ: Splicing factor proline and glutamine rich
CRMP4: Collapsin response mediator protein 4
VCP: Valosin-containing protein
ATGs: Autophagy-related proteins
NF-κB: Nuclear factor kappa B
Tregs: Regulatory T cells
ROS: Reactive oxygen species
APP: Amyloid precursor protein
RRIM: RIPK1-regulated inflammatory microglia
STING: Stimulator of interferon genes
hiPSCs: Human-induced pluripotent stem cells
SPT: Serine palmitoyltransferase
SPTLC: Serine palmitoyltransferase long chain base subunit
DCTN1: Dynactin subunit 1
